# Transcriptome and functional analysis reveals hybrid vigor for oil biosynthesis in oil palm

**DOI:** 10.1038/s41598-017-00438-8

**Published:** 2017-03-27

**Authors:** Jingjing Jin, Yanwei Sun, Jing Qu, Rahmad syah, Chin-Huat Lim, Yuzer Alfiko, Nur Estya Bte Rahman, Antonius Suwanto, Genhua Yue, Limsoon Wong, Nam-Hai Chua, Jian Ye

**Affiliations:** 1 Temasek Life Sciences Laboratory, National University of Singapore, 1 Research Link, 117604 NUS, Singapore; 2 Laboratory of Plant Molecular Biology, Rockefeller University, 1230 York Avenue, New York, NY 10021 USA; 3 School of Computing, National University of Singapore, 117417 NUS, Singapore; 4 China Tobacco Gene Research Center, Zhengzhou Tobacco Research Institute of CNTC, Zhengzhou, Henan 450001 China; 5 State Key laboratory of Plant Genomics, Institute of Microbiology, Chinese Academy of Sciences, Beijing, 100101 China; 6 R&D Department, Wilmar International Plantation, Palembang, Indonesia Biotech Lab, Wilmar International, Jakarta, Indonesia

## Abstract

Oil palm is the most productive oil crop in the world and composes 36% of the world production. However, the molecular mechanisms of hybrids vigor (or heterosis) between *Dura*, *Pisifera* and their hybrid progeny *Tenera* has not yet been well understood. Here we compared the temporal and spatial compositions of lipids and transcriptomes for two oil yielding organs mesocarp and endosperm from *Dura*, *Pisifera* and *Tenera*. Multiple lipid biosynthesis pathways are highly enriched in all non-additive expression pattern in endosperm, while cytokinine biosynthesis and cell cycle pathways are highly enriched both in endosperm and mesocarp. Compared with parental palms, the high oil content in *Tenera* was associated with much higher transcript levels of *EgWRI1*, homolog of *Arabidopsis thaliana* WRINKLED1. Among 338 identified genes in lipid synthesis, 207 (61%) has been identified to contain the WRI1 specific binding AW motif. We further functionally identified *EgWRI1*-*1*, one of three *EgWRI1* orthologs, by genetic complementation of the *Arabidopsis wri1* mutant. Ectopic expression of *EgWRI1*-*1* in plant produced dramatically increased seed mass and oil content, with oil profile changed. Our findings provide an explanation for *EgWRI1* as an important gene contributing hybrid vigor in lipid biosynthesis in oil palm.

## Introduction

Hybrid vigor, or heterosis, refers to the superior performance of hybrid progeny relative to their inbred parents^1–4^. Heterosis in plants of diverse species is associated with many superior agronomic characteristics, including larger plant stature, increased biomass, growth rate, grain yield, and tolerance to abiotic stresses. These heterosis is frequently found in cereal crops such as maize (*Zea mays*) and rice (*Oryza sativa*), mainly as hybrids, and many other crops including bread wheat (*Triticum aestivum*), upland cotton (*Gossypium hirsutum*), and few in oil crop e.g. canola or rape seed (*Brassica napus*), are grown as allopolyploids. Heterosis has been widely used by breeding program to improve crop yield and resistance significantly. Despite being widely exploited for many decades, the mechanisms underlying heterosis still remain poorly understood esp. for high energy content oil crop^5^. Most of the earlier efforts focused on genetic analysis of the modes of gene action. Many observations point to the likelihood that the diverse genetic and molecular mechanisms at a large number of genes are responsible for hybrid vigor in any heterotic parental pair. A few classical genetic concepts were previously considered to explain hybrid vigor and the most accepted hypothesis are dominance, the over-dominance and epistatic effects^6^. Recently, it was suggested that a combination of different genetic principles might best explain the mechanism of hybrid vigor^7,8^. However, identifying the specific molecular mechanisms that are responsible for the phenotypic differences between the hybrid and inbred parents remains a challenge, although successes in other plants have been achieved, such as the demonstration of heterosis in yield by the effects of a single gene *Single Flowering Truss* in tomato^9^.

In recent years, study of heterosis has been approached to the level of the gene products such as transcripts and the regulation of their expression. Available technologies have enabled addressing the biological concerns at the genomic scales, the proteome, the metabolome and especially transcriptome. Various mechanisms may be involved in RNA expression regulation including genetic or epigenetic factors, such as chromosomal structure, DNA sequence diversity, and DNA methylation^6^.

The use of hybrid have been highly promoted in oil crops yield including rape seed^10–12^, soybean^13^ and most important oil palm^14^. Oil palm (*Elaeis guineensis* Jacq) originates from tropical Africa and it was imported into South Asia where industrial plantations started about 100 years ago. Oil palm is now the most productive oil (triacylglycerol, TAG) crop in the world with an average oil yield of 5–7 tons per hectare^15^ and contributes about 36% of world oil production. In addition to triglycerides, palm oil also contains high levels of carotenoids and vitamin E, which are believed to counter the ravages of chronic diseases such as heart disease and cancer and to delay aging^15^.

Almost all modern, commercial planting material consists of *Tenera* palms by crossing of *Dura* and *Pisifera*. Crossing *Dura* and *Pisifera* to give the hybrid progeny fruit type improved partition of dry matter within the fruit, resulting in a more than 30% increase in oil yield per tree. With the progress of genome sequencing project for parental *Dura*^16^ and *Pisifera*^15^, it is possible to underpin the molecular mechanisms of hybrid vigor of *Tenera*. Therefore, this model will be used to understand heterosis in lipid biosynthesis for oil crop. A few efforts of genome-wide gene identification for oil biosynthesis have been done^14–19^. Despite its obvious scientific and economic interest, literature and molecular resources available for oil palm remain elusive, esp. for the molecular mechanism of hybrid vigor in oil yield is still scarce. Aside from the *SHELL* gene that developmentally controls cell type differentiation^20^, the heterosis in lipid biochemical synthesis and the molecular clues are largely unknown. Furthermore, the chemical and biochemical basis of oil yield heterosis need to be further characterized.

Presently, knowledge of TAG accumulation in plants is mostly based on studies of oil seeds. Sucrose as the main source of carbon for storage oil synthesis in higher plants, is converted to pyruvate via glycolysis in non-green tissues. Pyruvate is the main precursor for the acetyl-CoA molecules destined to fatty acid synthesis. Plastid pyruvate kinase (PK), pyruvate dehydrogenase (PDH), and acetyl-CoA carboxylase are considered as key enzymes for fatty acid synthesis and are regulated by transcription factors in oil seeds, including WRINKLED1 (WRI1)^21–26^.

We have recently identified some candidate gene responsible for tree height in oil palm^27^. We also sequenced *Dura* genome and did resequencing on elites’ trees^16^. With the whole genome sequence availability, we here try to globally survey the transcriptome of the two inbred lines *Dura* and *Pisifera* in comparison to their F1-progeny *Tenera* in an unbiased approach via RNA-seq. To identify features specific to high oil production in non-seed tissues such as oil palm mesocarp, we first identified the key lipid biosynthesis stage and generated several billion reads for the mesocarp. At the same time, we did parallel work for seed part of the endosperm. By choosing the particular stages, we intended to identify transcriptional patterns and regulators possibly determining the processes leading to heterosis for the lipid traits. Moreover, we carried out transgenic analysis to identify candidate key genes to be responsible for hybrid vigor in oil biosynthesis of oil palm. The focus of this temporal and comparative analysis was to gain insight into factors responsible for this dramatic difference in carbon partitioning. In addition, a better understanding of oil accumulation in fruits may present strategies for engineering oil accumulation in other vegetative tissues.

## Results

### Hybrid vigor in lipid biosynthesis in oil palm

**Figure 1:**
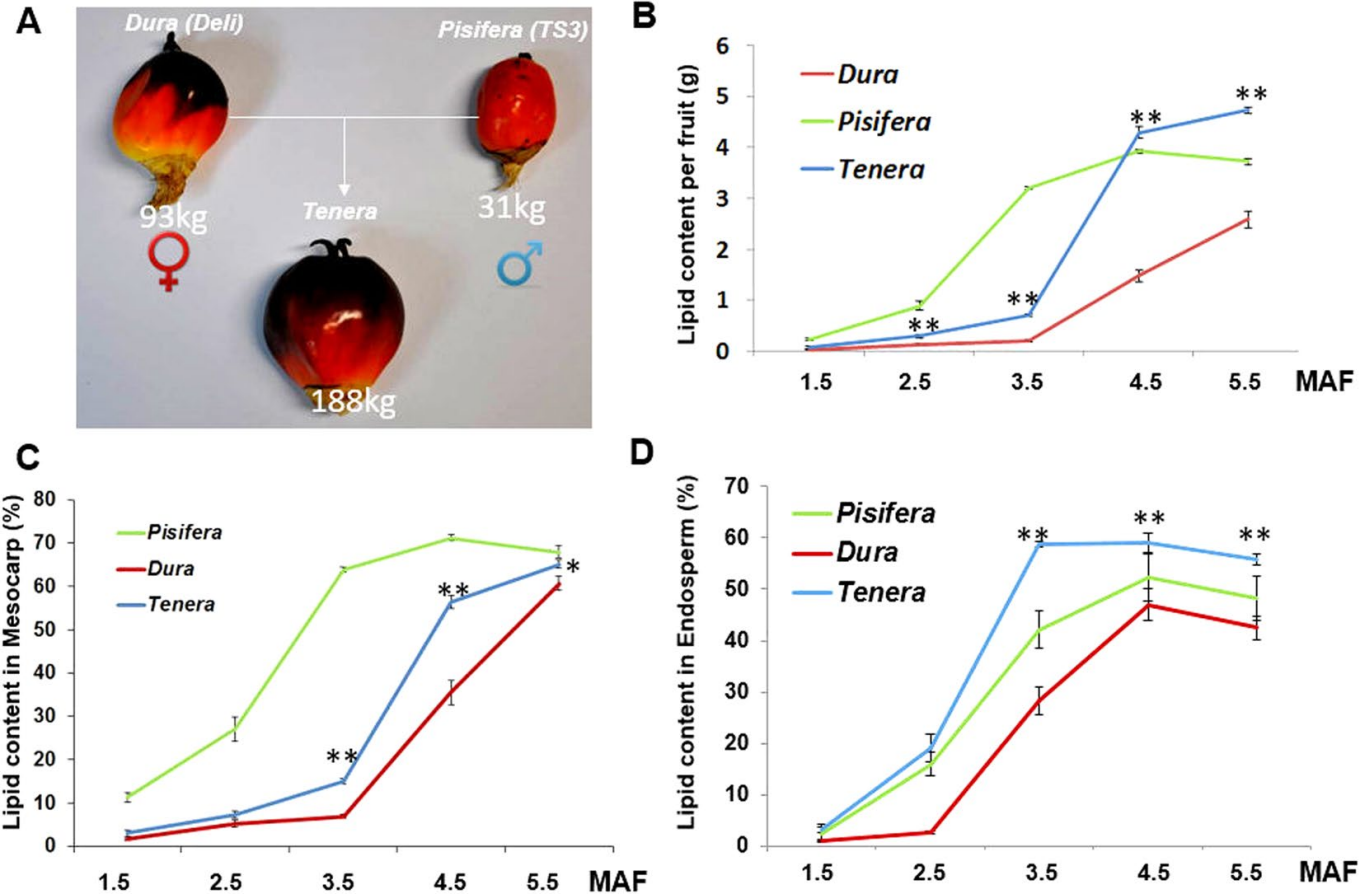
Heterosis in lipid biosynthesis observed in *Tenera* crossed by *Pisifera* and *Dura*. (**A**) Fruits of three African oil palm (*Elaeis guineensis*) varieties, *Dura* (Deli), *Pisifera* (TS3), and the hybrid progeny tree (#53). Mean total fertile fruit weight per year (kilogram) per tree was recorded below of each variety. Yield data of 4^th^ and 5^th^ years after planting were analyzed. (**B**) Lipid content for fruit of *Dura*, *Pisifera* and *Tenera* during fruit development stages. Fruit developmental stages were recorded as Months after Fertilization, MAF. Values are mean total lipid amount of mesocarp and endosperm (gram) ± SD (n = 6) (Student’s *t*-test). Asterisks in all panels indicate significant differences between *Tenera* and *Dura* (*P < 0.05; **P < 0.01). (**C**) Mesocarp lipid content of *Dura*, *Pisifera* and *Tenera*. Values are mean total lipid amount of mesocarp (% dry weight) ± SD (n = 3) (Student’s *t*-test). (**D**) Endosperm lipid content of *Dura*, *Pisifera* and *Tenera*. Values are mean total lipid amount of endosperm (% dry weight) ± SD (n = 3) (Student’s *t*-test).

As an initial step to know whether there is hybrid vigor of lipid biosynthesis in oil palm, we firstly performed the analysis for the fertile fruit yield per tree per year. Figure 1A indicates that fruit biomass amount has clear heterosis over both of parents as shown by a much higher fruit biomass amount produced in *Tenera* than parental oil palms *Dura* and *Pisifera*. Due to extremely low fertile fruit ratio for *Pisifera* (only less 10% VS 70% for *Dura* and *Tenera*), the low fruit yield palm *Pisifera* is not commercially used in oil palm plantation except to provide pollen for generating the hybrid progeny *Tenera*. Further analysis on key lipid traits, for example lipid content per fruit (Fig. 1B), lipid content in mesocarp and endosperm (Fig. 1C and D), strongly suggest that *Tenera* has heterosis in lipid related traits, including heterosis over both of parents in trait of lipid yield per fruit, and heterosis over previous used variety *Dura* in traits of lipids content in both lipid yield tissues (mesocarp and endosperm) as indicated in final mature fruit stages (5.5 months after fertilization). Mesocarp of *Pisifera* commences rapid lipid biosynthesis and accumulation at very early stage, as highly as 10% of total lipid content at 1.5 months after fertilization (MAF), 2 months ahead of *Tenera*, which is one month ahead of *Dura* at 4.5 MAF (Fig. 1C). Microscopy of 4.5 MAF oil palm mesocarp showed, most notably, cells with numerous and more obvious oil droplets were observed in *Tenera* than in *Dura* (Supplementary Figure S1A and B). There is no obvious cell size difference between mesocarp of *Dura* and *Tenera* (Supplementary Figure S1A and B).

Similarly, heterosis is also found in endosperm. Earlier rapid lipid biosynthesis is taken place in endosperm of *Tenera* and lipid content in endosperm was also found to be much higher in *Tenera* than in *Dura* (Fig. 1D). Consistent with a lipid content drop in late stage of endosperm, the secondary cell wall was enhanced (Supplementary Figure S1C), as compared with early stage, indicating carbon redistribution in endosperm in later developing stage (Supplementary Figure S1D). By further detailed dissecting of 5 different stages ripening fruits, key periods for rapid lipid biosynthesis were identified as 3.5 MAF for mesocarp and 2.5 MAF for endosperm (Fig. 1C and D). We then used RNAseq samples of these five stages from *Dura* and *Tenera* to further underpin how lipid traits heterosis happens in oil palm hybrid.

### Transcriptome profiling analysis

In this study, 24 samples, around 20 million RNA seq reads per sample, with 101 bp pair-end, were obtained for 5 stages of three oil palm mesocarp and 3 stages of three oil palm endosperms respectively (Supplementary Dataset S1). After de-novo assembly of these reads, 96,062 isoforms and 59,078 unigenes, with total 106,097,832 bp and GC content of 44.43% were identified. The N50 is 1,884 bps. 41.7% of genes were highly similar (BLASTX E-value < 1e-3) to *Arabidopsis thaliana* proteins. Contigs were annotated on the basis of *Arabidopsis thaliana* proteins since it is by far the best-annotated proteome among the plant kingdom.

**Figure 2:**
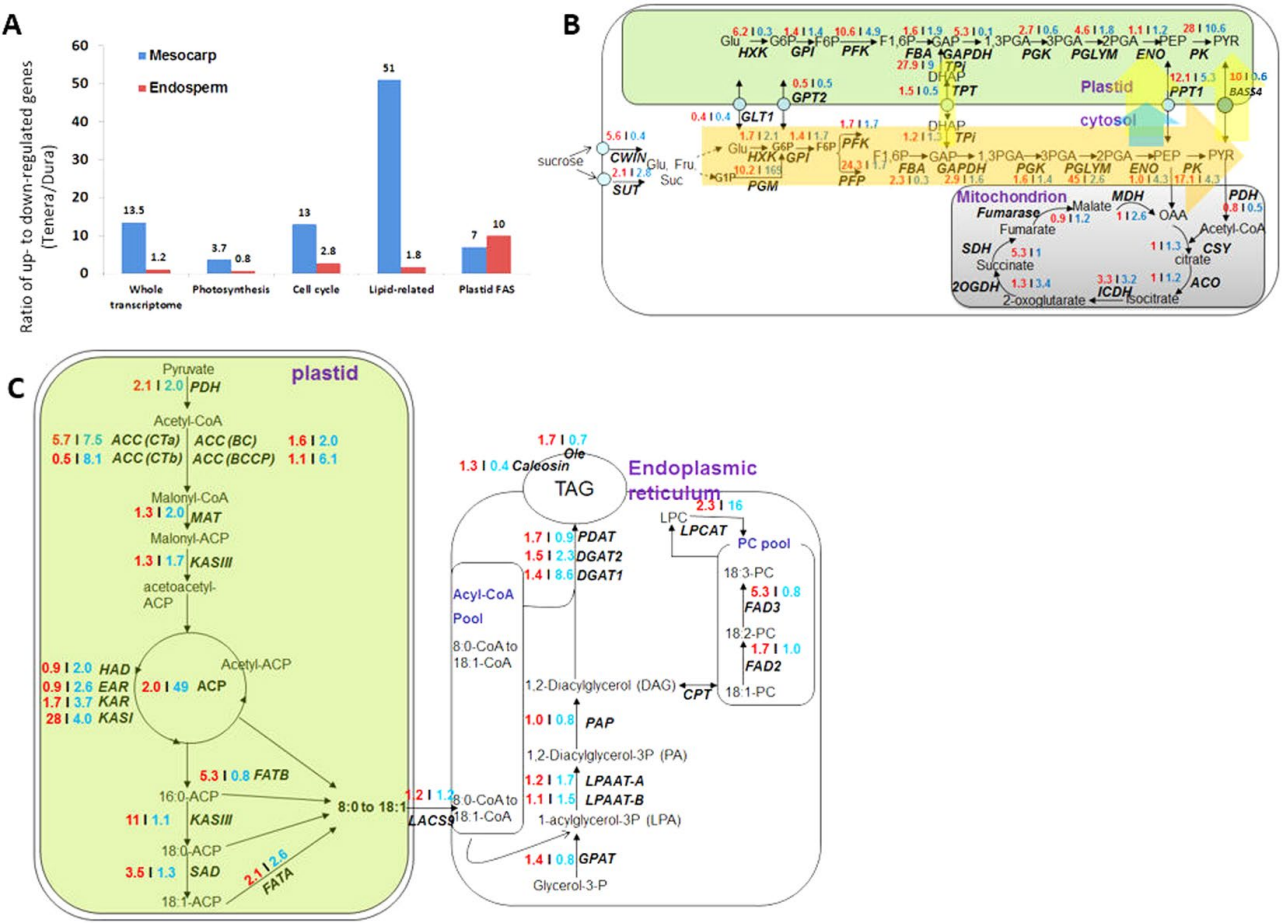
Transcriptome pattern differs between two oil biosynthesis tissues in oil palm. (**A**) The ratio of up- to down-regulated genes when compared the transcription level of *Tenera* against *Dura* in mesocarp and endosperm. The gene expression level of their respective key stage (2.5 MAF for endosperm, 3.5 MAF for mesocarp) were used to compare. Assumed at least 2 folds change as up- or down-regulated genes. Whole transcriptome and some specific categorized pathways are listed out. Plastid FAS refers to the pathway of fatty acid synthesis in plastid. Noticed that genes of lipid related or plastid FAS are highly upregulated in mesocarp and endosperm of *Tenera* respectively. (**B**) Transcripts patterns for enzymes involved in glysolysis reactions for *Dura* and *Tenera*. Values in red indicate the fold increase in reads of mesocarp of *Tenera* to that of Dura at 3.5 MAF. Values in blue indicate the fold increase in reads of endosperm of Tenera to that of Dura at 2.5 MAF. Samples collected from different tissues for *Dura*, *Pisifera* and *Tenera* (#53) were used for transcriptome analysis. Orange arrow indicates the cytosolic glycolytic process is the major carbon flow increased in *Tenera* compared with *Dura* both in mesocarp and endosperm, as deduced by the fold increase in reads. Light yellow arrows indicate the major increased cytosol-to-plastid carbon flow for mesocarp. Light blue arrow indicates the major increased cytosol-to-plastid carbon flow for endosperm. (**C**) Transcripts patterns for enzymes involved in plastidial and extraplasidial reactions for *Dura* and *Tenera*. Values in red indicate the fold increase in reads of mesocarp of *Tenera* to that of *Dura* at 3.5 MAF. Values in blue indicate the fold increase in reads of endosperm of *Tenera* to that of *Dura* at 2.5 MAF. Samples collected from different tissues for *Dura*, *Pisifera* and *Tenera* (#53) were used for transcriptome analysis. Reads for enzymes with multiple isoforms were summed. For details on abbreviations, annotations, and transcriptome levels at each samples, see Dataset S1. 16:0, palmitic acid; 18:0, stearic acid; 18:1, oleic acid, 18:2, Linoleic acid; 18:3, linolenic acid. For details on abbreviations, annotations, and transcription levels at each stage, see Supplemental Dataset S1.

We have identified the key periods for rapid lipid biosynthesis as 3.5 MAF for mesocarp and 2.5 MAF for endosperm (Fig. 1). Here we focused on these two stages for comparative transcriptome analysis to understand the molecular basis of lipid heterosis in oil palm. Astonish difference was identified between mesocarps of *Dura* and *Tenera*, as there is a much higher ratio (13.5:1) of upregulated expression gene than downregulated expression gene (Fig. 2A), from whole transcriptome view as a basis of 59,078 genes in total. Intriguingly, compared with other categories such as photosynthesis and cell cycle, it is clear that the lipid-related categorized genes are highly enriched in unregulated genes as indicated by the ratio of up-regulated to down-regulated genes as 51, giving a good explanation why *Tenera* has heterosis in lipid related traits.

### Lipid biosynthesis pathway genes exhibit hybrid vigor in endosperm but not significantly in mesocarp

To determine relative expression levels of genes in hybrids as compared to their parental inbred lines and to identify genes displaying expression levels that differ from additivity, eight gene expression classes were defined (Table 1) based on similar classifications published previously^8,28,29^. First, for any given gene, differential expression values in the hybrid relative to the parental inbred lines displaying the lower (low-parent, LP) and higher (high-parent, HP) expression levels, were estimated by fold change (Table 1). According to this classification scheme, 10,801 (endosperm) or 11,574 (mesocarp) genes were expressed in hybrid at least higher than one of the parental values (class 2, 5, 6, Table 1). Class 4 represents the largest class, containing 70% genes of the total that do not exhibit differential expression among the parents and hybrids. Furthermore, 1,107 (endosperm) and 230 (mesocarp) genes exhibited low-parent expression levels in the hybrid (class 7 and 8).

**Table 1:** Gene number in each group.

Class	Expression pattern	Endosperm		Mesocarp	
		Total gene num	Non-additive gene num	Total gene num	Non-additive gene num
1	LP < H < HP	459	223	404	181
2	LP < H = HP	8,040	8,040	6,904	6,904
3	LP = H < HP	5,211	0	4512	0
4	LP = H = HP	41,500	6,015	42,358	13,082
5	LP < HP < H	2,478	2,478	3,794	3,794
6	LP = HP < H	283	283	876	876
7	H < LP < HP	464	0	116	0
8	H < LP = HP	643	0	114	0
	Total	59,078	17,039	59,078	24,837

LP: low parent, H: hybrid (Tenera), HP: high parent; <, > means at least two fold change (FC); = means the fold change is small than 2.

Within these classes, a subset of genes displayed nonadditive gene expression, i.e., expression levels in a hybrid that were significantly different from the average of the parental values (MPV: mid-parent value). A significant higher portion of genes in mesocarp showed nonadditive expression pattern, indicating a remarkable expression changes have taken place in mesocarp than seed organ endosperm (Table 1). We further analyzed whether lipid biosynthesis pathways are enriched in nonadditive expression gene by whole genome analysis. Though there is no enrichment on lipid biosynthesis in all non-additive genes showed in Table 1, but multiple lipid biosynthesis pathways are found to be highly enriched in endosperm (Supplementary Table S1), but not so significantly in mesocarp (Supplementary Table S2).

**Figure 3:**
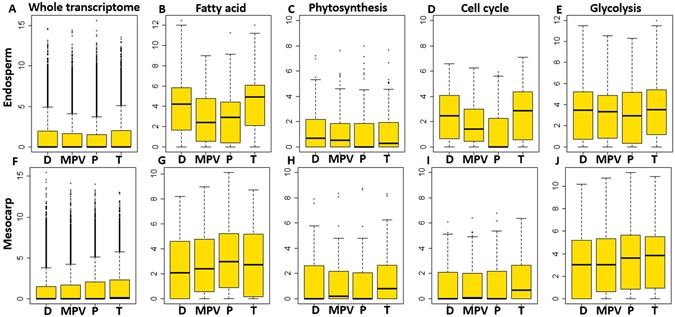
Heterosis, additive and non-additive gene expression. The midparent value (MPV) is equal to (Dura + Pisifera)/2, respectively. The gene expression based on the whole transcriptome and each pathways in Endosperm (**A**–**E**) or mesocarp (**F**–**J**) were boxploted. D, P, T in x-axis represents Dura, Pisifera and Tenera. Y-axis represents normalized expression level.

A detailed analysis between additive and nonadditive gene expression based on whole genome or individual pathway data generated similar conclusion for both endosperm and mesocarp (significant difference between Tenera and MPV by t-test analysis) (Fig. 3). Interestingly, cytokinine biosynthesis and cell cycle genes pathways are highly non-additive or displayed better than parents in both endosperm and mesocarp, explaining the confirmed rapid developing phenotype in hybrid by hyperactivity of cell proliferation phytohormone cytokinine (Supplementary Tables S1 and S2 and Fig. 3). Furthermore, photosynthesis genes exhibited hybrid vigor in mesocarp, whereas endosperm exhibited an even lower than MPV level in a hybrid (Fig. 3).

### Expression pattern of genes involved in glycolysis and fatty acid biosynthesis

The expression of glycolysis genes is highly upregulated in mesocarp of *Tenera* against these of *Dura*. As shown in Fig. 2B, glycolysis, esp. the cytosolic pathway genes are highly expressed (Supplementary Figure S2). The carbon flow enhancing by presumed sucrose importing into developing fruits from photosynthetic organs (such as leaves), takes the highway in cytosol to biosynthesis more PEP and pyruvate, which are further served as substrates and transported into plastid for plastidial fatty acid synthesis. The genes encoding the cytosol-to-plastid transporters, which located in plastid membrane, POLYPRENYLTRANSFERASE 1 (PPT1) and BILE ACID: SODIUM SYMPORTER FAMILY PROTEIN 4 (BASS4) starting from the endosperm part, a relatively higher portion of genes are upregulated in fatty acid biosynthesis compared with the whole transcriptome level (1.8 VS 1.2). Only two subcategories Plastidial Fatty Acid Synthesis and extra Plastidial Phospholipid Synthesis are highly enriched (Fig. 2A and Supplementary Figure S4). On the contrary in mitochondria, the expression of genes encoded for enzymes in citric acid cycle, sharing the same substrate (pyruvate) with lipid biosynthesis, are at a similar level between *Dura* and *Tenera* on two tissues (Fig. 2B, Supplementary Figure S2).

Including the first enzyme complex of conversion pyruvate to fatty acids, PDH, 12 genes encoded for fatty acid synthesis (FAS) enzymes were, on average, 8-fold higher in endosperm of *Tenera* than that of *Dura* (Fig. 2C, Supplementary Figure S3). These expression data together with lipid content data indicated a closely coordinated expression during oil synthesis in seed tissue, and a pattern also noted in *Arabidopsis thaliana* and other seeds. The largest individual difference was noted for ketoacryl-acyl carrier protein (ACP), for which the expression was about 50-fold higher in *Tenera* relative to *Dura*. Contrary to highly expression of FAS related genes in endosperm, the level of FAS transcripts only mildly increased in *Tenera* than in *Dura* in mesocarp, except ketoacyl ACP synthase I (*KASI*), which encodes the key enzyme for fatty acid elongation, with an increase of 28-fold in *Tenera* (Fig. 2C). The mild increased expression of FAS genes is correlated with moderate lipid content shown in Fig. 1C. In contrast to a weak increase in FAS, the genes encoded for enzymes to determinate fatty acid profile/composition were, on average, 5-fold higher in mesocarp of *Tenera* than that of *Dura*, esp. for the ketoacyl ACP synthase II (*KASII*), which encodes the enzyme to convert C16 to C18, with an increase of 11-fold in *Tenera*. Since the expression changes were found in key genes for fatty acid composition determination, we deduced a higher C18:C16 ratio in *Tenera*. Gas chromatographic analysis showed that a much higher C18:C16 ratio in mesocarp of *Tenera* (1.60) than that of in *Dura* (1.28), consistent with the much higher *KASII* expression level found in mesocarp of *Tenera* (Fig. 2C). The desaturated to saturated fatty acid ratio was also increased in mesocarp of *Tenera* (1.23) than that of in *Dura* (1.0) (Supplementary Table S4). The higher C18:C16 level and higher desaturated fatty acid are the two key breeding targets for healthier palm oil by reducing “bad lipid” saturated palmitic acid (C16:0) level. The highly expression of FATTY ACID DESATURASE 3 (*FAD3*), encoding enzyme to synthesis α-linolenic acid C18:3 and longer fatty acid, are highly expressed in *Tenera*, fitting well with higher C18:3 and further derivate fatty acids in *Tenera* (3.6% Vs 0.7% in *Dura*) (Supplementary Table S4). In sharp contrast to the expression patterns for plastidial fatty acid enzymes, the TAG assembly in most enzymes showed similar level in both of endosperm and mesocarp of *Tenera* and *Dura*, except the two DAG:acyl-CoA acyltransferase (DGAT1 and DGAT2) that catalyse the last (acyl-CoA dependent) acylation step to TAG, with 8.6-fold and 2.3-fold of *Dura* in endosperm respectively (Fig. 2C).

### PC-Related Enzymes Showed heterosis between *Tenera* and *Dura*

Beside of the plastidial fatty acid and glycolysis pathways, the Endoplasmic reticulum (ER) membrane based polar phosphatidylchoilne (PC) synthesis pathway was also of highly ectopic expression in *Tenera* (Fig. 3, Supplementary Table S5 and Supplementary Figure S4). Several studies have demonstrated the importance of PC metabolism and acyl editing in the process of TAG assembly, which proceed via either the methylation or nucleotide pathway. Bourgis *et al*. have shown that PC-Related enzymes show higher transcriptome level than the date palm major for sugar biosynthesis^18^, which suggest that PC-related enzymes might have some important functions on carbon partitioning. Consistently, by using our transcriptome data, we found out that most of the genes encoding PC-related enzymes were higher in *Tenera*, as compared to *Dura*, especially in mesocarp, such as choline kinase (CK), choline-phosphate cytidylyltransferase (CCT) and long-chain acyl-CoA synthesis (LACS) (Supplementary Figure S4). Furthermore, the mutation of phosphatidylcholine:diacylglycerolcholine phosphotransferase (PDCT) gene in *Arabidopsis* reduced polyunsaturated fatty acids linoleic acid 18:2 and 18:3 level accompanied with increased 18:1 level. We observed the enhanced expression of two *PDCT* genes (*PDCT1* and *PDCT2*) in *Tenera*, which may explain the reverse trend of fatty acid composition change (higher poly- VS lower monosaturated fatty acid), compared with that of *Dura* in both mesocarp and endosperm (Supplementary Figure S2, Supplementary Tables S3 and S4). Hence, our data suggest that phospholipid and methylation pathway may play important roles in lipid heterosis between *Tenera* and *Dura*.

In summary, the expression pattern for most of the genes in lipid biosynthesis pathway is consistent with metabolite profiling analysis. Enhanced expression of genes in glycolysis and lipid biosynthesis pathways may be one of the fundamental reasons for the lipid heterosis of *Tenera* over its parental.

### WRI1 transcription factors genes upregulated in *Tenera* than parental palms

**Figure 4:**
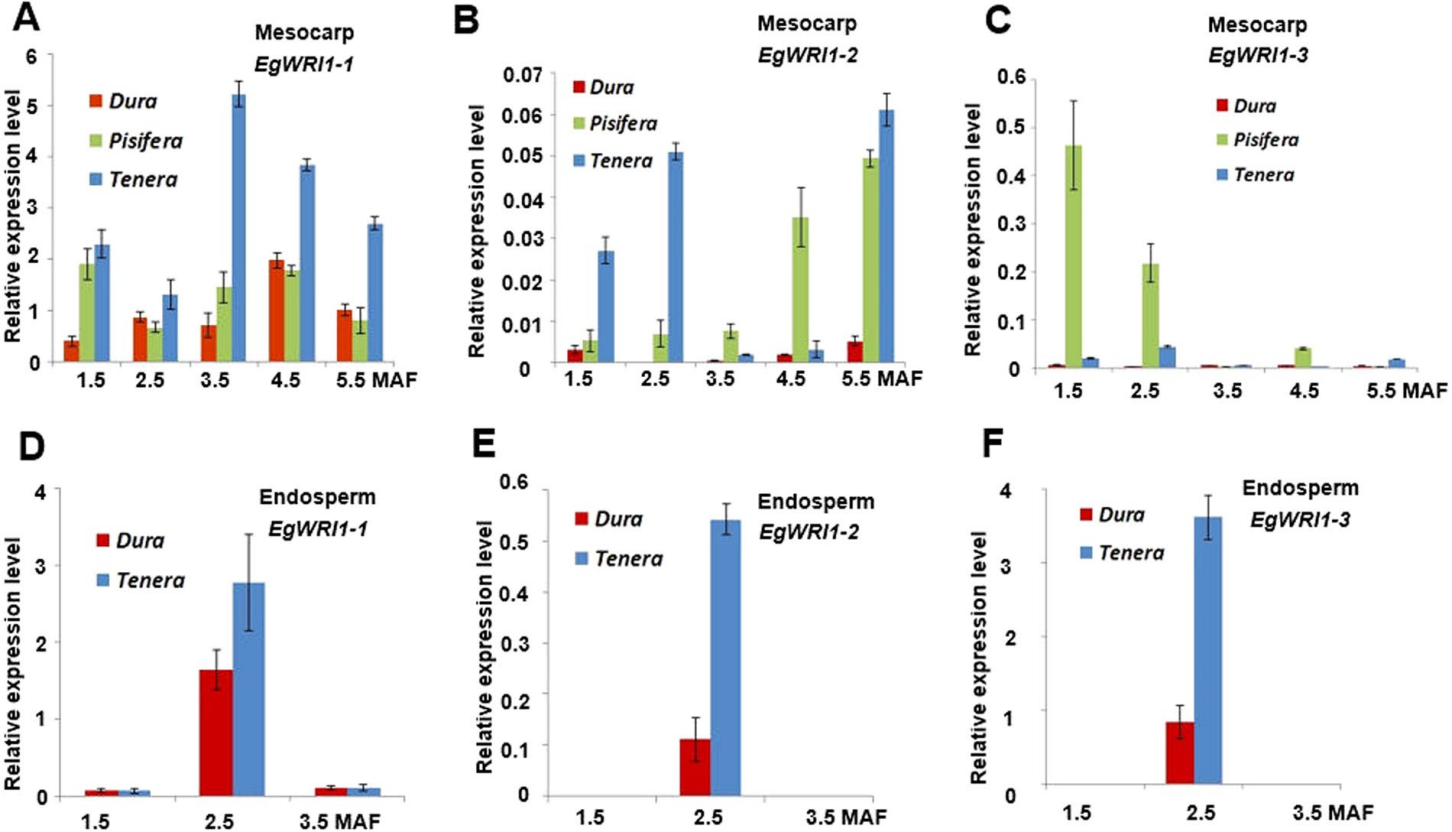
Relative expression of three *WRI1* genes (*EgWRI1*-*1*, *-2*, *-3*) in mesocarp (**A**–**C**) and endosperm (**D**–**F**). The data was presented as means ± standard deviations (n = 3).

Since there are many lipid biosynthesis genes upregulated in both tissues of *Tenera* compared with *Dura*, we suspected that some master regulators such as transcriptional factor may be involved in the transcriptional regulation of lipid heterosis. Three paralogs of the WRINKLED1 (WRI1) transcription factor were identified from our own and other published transcriptome data^14,18^. Here, we performed real-time quantitative RT-PCR to analyse the expression profile of three *WRI1* genes in various tissues at different developing stages by designing gene specific primers shared by *EgWRI1*-*1* and *EgWRI1*-*2*. As shown in Fig. 4, the expression of all there *WRI1* coincided well with the oil biosynthesis in mesocarp and endosperm (Fig. 1). In general, all three WRI1 genes showed higher expression level in endosperm than in mesocarp. *EgWRI1*-*1* gene expression is the highest, specifically at 3.5 MAF mesocarp and 2.5 MAF endosperm of *Tenera*. The expression level of *EgWRI1*-*2* is only 1% of that of *EgWRI1*-*1* in *Tenera*. *EgWRI1*-*3* is highly expressed in early stages (1.5 to 2.5 MAF) of mesocarp of *Pisifera*, which suggested that the early rapid lipid biosynthesis accumulation may due to the early expression of *EgWRI1*-*1* and *EgWRI1*-*3* in mesocarp of *Pisifera* (Fig. 4). In almost all the stages we tested, three WRI1 genes are higher expression in *Tenera* than *Dura*, suggesting a strong transcription activity of this lipid biosynthesis master transcription factor may be one of the reasons to explain highly expressed lipid biosynthesis pathway genes, by which lipid heterosis took place in *Tenera*, both in mesocarp and endosperm tissues. The mechanisms why all three WRI1 genes are highly expressed in *Tenera* are still unknown. However, several groups have found that WRI1 can use AW motif to regulate other lipid related genes^19,22,23^. Among 338 identified genes in lipid synthesis by our transcriptome data, 207 (61%) has shown to contain WRI1 specific binding AW motif, same with previous findings^22^. This may also be one of the reasons to explain the important functions of WRI1 in oil yield of oil palm (Supplementary Dataset S1). In addition, we also tried to find some other upstream transcription factor genes, which might be correlation with WRI1’s expression, such as *LEAFY COTYLEDON1* and *PICKLE*, but failed.

### WR1-1 and WRI-3 increase oil content and quality in transgenic plants

**Figure 5:**
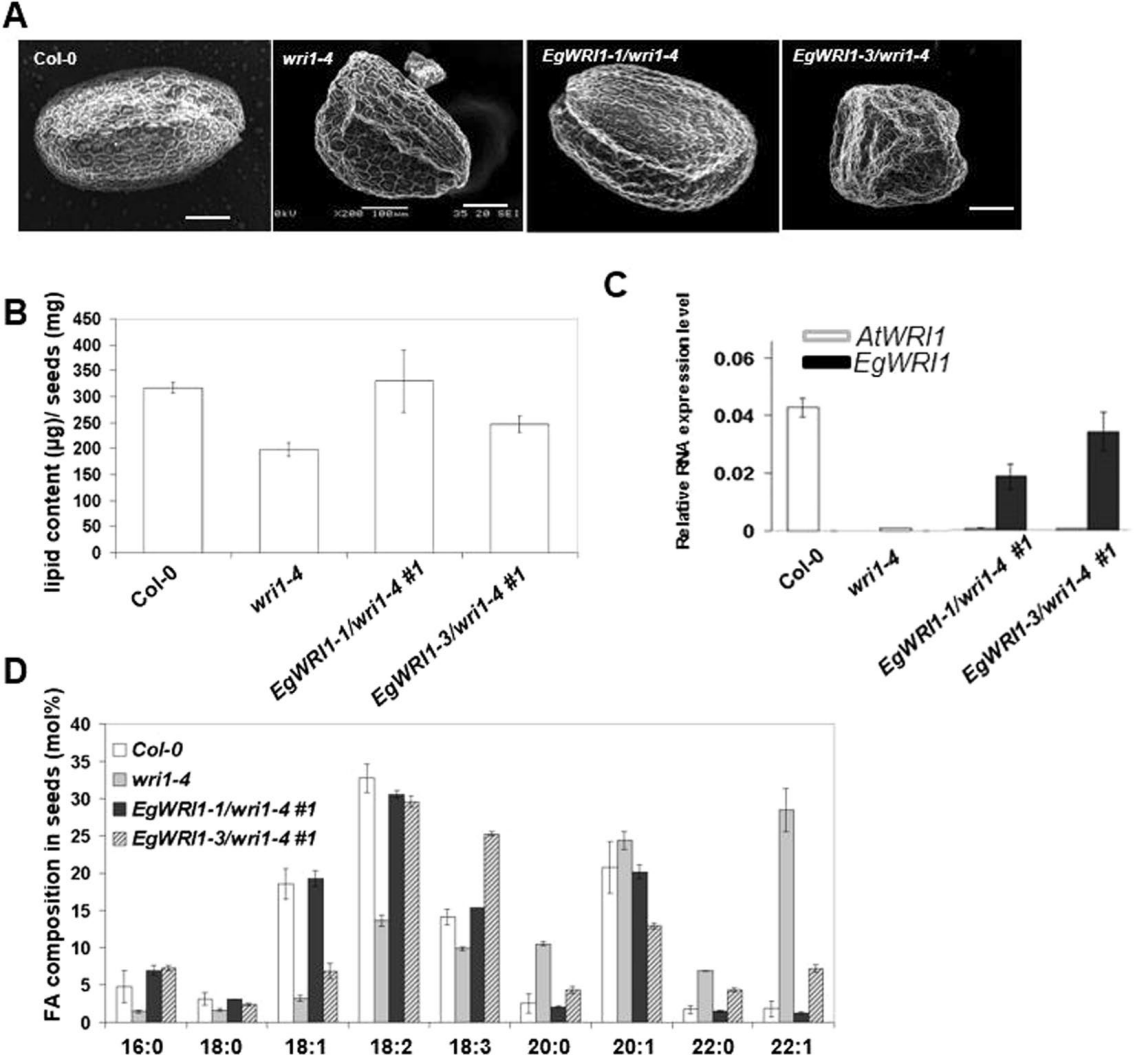
Functional analysis of *EgWRI1*-*1 and EgWRI1*-*3* in *Arabidopsiswri1*-*4* mutant background. (**A**) Images of mature seeds of the wild type (ecotype Columbia-0 [Col-0]), mutant (*wri1*-*4*), complemented mutant with oil palm *Oleosin* promoter (*EgOle*:*EgWRI1*-*1*/*wri1*-*4*), and non-complemented mutant (*EgOle*:*EgWRI1*-*3*/*wri1*-*4*). Size bar: 100 µm. (**B**–**D**) Analysis of seed oil traits of Col-0, *wri1*-*4*, *wri1*-*4* lines complemented with similar expression level of *EgWRI1*-*1* or *EgWRI1*-*3*. Seed oil content (**B**) and fatty acid (FA) composition (**D**) were analyzed. (**C**) The expression level of both *AtWRI1* and oil palm *WRI1* (*EgWRI1*/*EgWRI3*) in complementary lines were measured by qRT-PCR. Error bars correspond to the SD calculated from three technical replicates per pool of 100 seeds.

To further investigate the function of WRI1 paralogs *in planta*, complementary experiments were carried out in *Arabidopsis*. Due to the high sequence similarity between *EgWRI1*-*1* and *EgWRI1*-*2*, and EgWRI1-2 was further exclude due to much lower expression level in various tissues and developing stages, we chose *EgWRI1*-*1* and *EgWRI1*-*3* to do transgenic work in *Arabidopsis*. Homozygous *Arabidopsiswri1*-*4* mutants were transformed with *EgWRI1*-*1* or *EgWRI1*-*3* cDNA^21^, the expression of which was driven by the seed-specific oil palm *Oleosin* promoter. For each construct, 5 independent primary transformants were selected and propagated; the progeny of T3 lines were subjected to detailed analyses. A microscopic observation of mature dry seeds showed a complete reversion of the wrinkled seed phenotype usually observed in the *wri1*-*4* mutant background (Fig. 5A). Fatty acid analyses confirmed the ability of only *EgWRI1*-*1* but not *EgWRI1*-*3* to restore the defect in not only fatty acid quantity but also fatty acid profile previously described in *wri1*-*4* seeds (Fig. 5B,D) with an even lower transgene expression level than *EgWRI1*-*3* (Fig. 5C).

**Figure 6:**
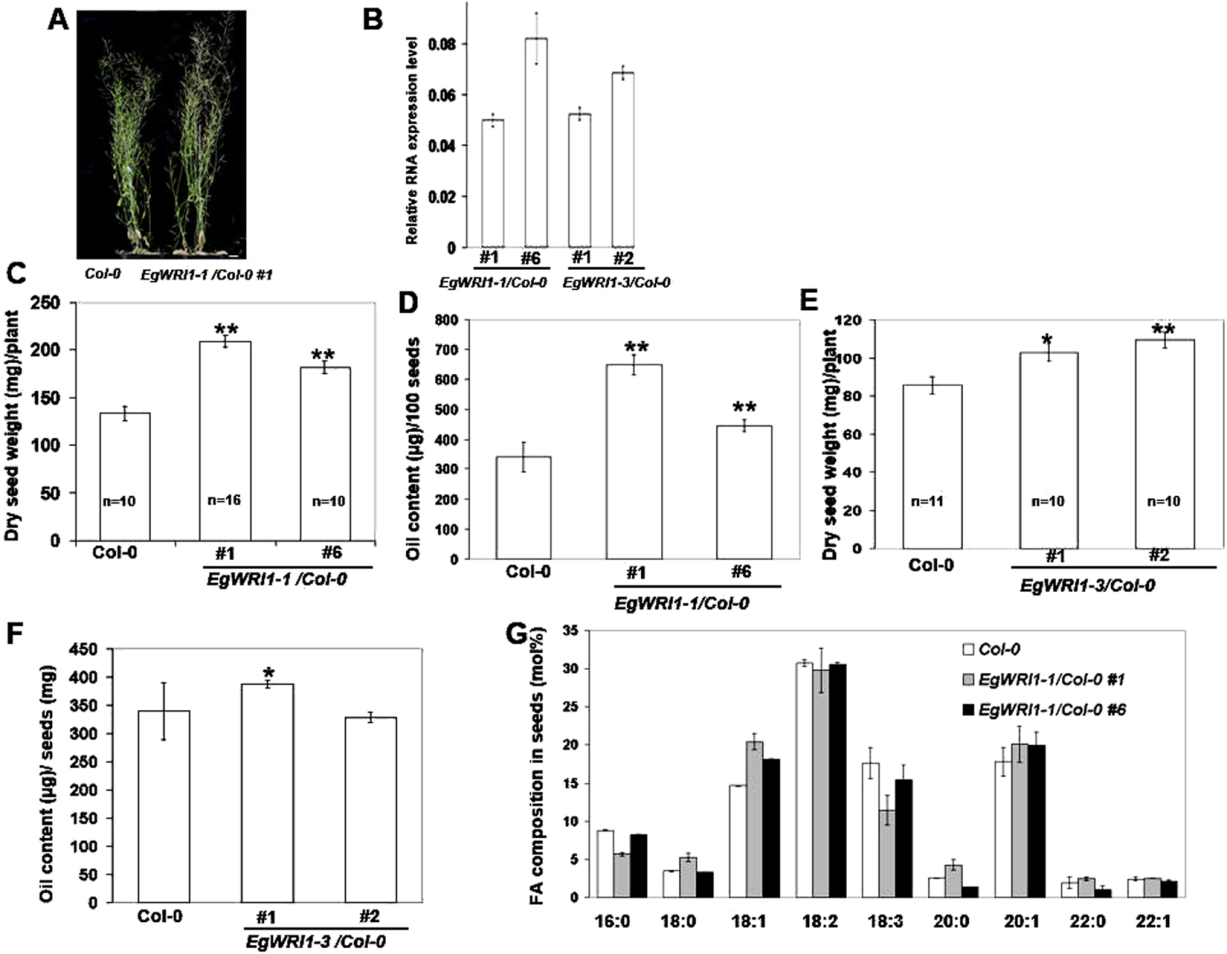
Functional analysis of *EgWRI1*-*1 and EgWRI1*-*3* in WT *Arabidopsis* background. (**A**) Images of mature plants of the wild type (Col-0) and *EgWRI1*-*1* expression line Size bar: 10 mm.(**B**) The expression level of EgWRI1-1 or EgWRI1-3 in seeds of overexpression *Arabidopsis* lines.(**C** and **E**) Dry seed weight of each plant for Col-0, two *EgWRI1*-*1* expression lines (**C**) and two *EgWRI1*-*3* expression lines (**E**). Plants number used was indicated. (**D** and **F**) Seed oil content for Col-0, two *EgWRI1*-*1* expression lines (**D**) and two *EgWRI1*-*3* expression lines (**F**). (**G**) Fatty Acid (FA) composition was analyzed for Col-0 and two *EgWRI1*-*1* expression lines.

We further generated transgenic *Arabidopsis* plants at WT background which expressed either *EgWRI1*-*1* or *EgWRI1*-*3* to test whether ectopic expression of these genes can produce more lipid than WT plants. Figure 6 showed that the ectopic expression of *EgWRI1*-*1* not only increase oil content but also produce more biomass and dry seed weight per plant, while EgWRI1-3 showed much weaker effect on oil yield increasing. The ectopic of *EgWRI1*-*1* also changed seed oil profile, as a significantly higher level of oleic acid level were observed in *EgWR11*-*1* overexpression lines. No effect of the expression of *EgWRI1*-*3* on fatty acid composition has been found in *EgWRI1*-*3* overexpression lines (Supplementary Figure S5).

## Discussion

Oil palm is the most productive oil-bearing crop. Over-represented genes in lipids metabolism and glycolysis are expressed differentially in mesocarp and endosperm of *Tenera*, accounting for the lipid heterosis and the different properties of palm mesocarp and endosperm oils. Due to non-additive variation, the heterosis observed in this research can be various in different combinations of parents.

Although heterosis has been widely exploited in plant breeding and plays an important role in agriculture^30–33^, the molecular and genetic mechanisms underlying the phenomenon remain poorly understood. Differential gene expression between a hybrid and its parents may be associated with heterosis. Hence, in this study, we used RNA-seq to compare the temporal and spatial change of lipids compositions and transcriptomes during the development of oil palm fruits for oil palm hybrid *Tenera* and its parents *Dura* and *Pisifera*. In comparison between *Tenera* and *Dura*, a much higher ratio (13.5:1) of up-regulated to down-regulated expression gene was observed in this transcriptome data, implying that the increased efficiency of a subset of transcripts were responsible for the heterosis between *Tenera* and its parental lines.

### Comparison of significant differential gene with QTLs for oil yield of oil palm

QTL provide links between genotype and phenotype for complex traits, and QTL analysis had been widely used to study heterosis in other crops^7,34^. These significant differential genes between *Tenera* and its parents are derived from the heterozygosis of the combined hybrid genomes and may be potentially associated with phenotypic changes in the hybrid. Although QTL analysis is still very limited because of the narrow number of markers^35,36^, We are still able to identify single nucleotide polymorphism (SNP) based on our sequencing data. After mapping SNPs to gene locus, a lot of allele specific genes were identified (Supplementary Figure S6A). If we focus on the fatty acid related groups, the portion is larger (Supplementary Figure S6B), implying that lipid heterosis in *Tenera* may be largely due to dominance, especially on the difference in allele frequency between the parental lines.

Marker-assisted breeding is a powerful tool for shortening the breeding generation time. Several papers have described the important traits with molecular markers development and application in oil palm breeding program^27,37^. These identified genes shown non-addictive effect in this report could be further used to develop molecular markers for oil traits mapping and elite tree characterization.

### Some other genes also exhibit hybrid vigor in Tenera

WRI1 genes are not supposed to control TAG assembly steps. However, DGAT1 and DGAT2 are highly expressed in endosperm of Tenera, correlated with higher expression of WRI1 genes in out studies. By generating transgenic Arabidopsis thaliana plants to express EgDGAT1 and EgDGAT2, DGAT1 ectopic expression in Arabidopsis thaliana can produce higher oil content than WT control (Supplementary Figure S7). Three acyl-acyl carrier protein thioesterase paralogs (EgFATB1, EgFATB2 and EgFATB3) have been reported to increase not only oil yield but also oil profile. We generated transgenic plants to express EgFATB1 or EgFATB2. Instead of increase oil yield, we observed a reduction of plant biomass by expression of EgFATB1 (Supplementary Figure S8). All saturated fatty acid composition (C16:0, C18:0, C20:0 and C22:0) are increased in EgFATB1 and EgFATB2, indicating both of them are important for saturated fatty acid biosynthesis and are good candidates to be manipulated to improve oil trait in the future.

### Networking but not dominance genes contributes to heterosis

Over-representation of additive expression in heterosis, the additive distribution of transcriptome in elite hybrids, and the low level or additively of intermediates suggest that there is an additively balance network regulating the heterosis between hybird and its parental lines. Fu *et al*. found that the germination-related hormone signal transduction, the abscisic acid and gibberellin regulation networks may contribute seed heterosis in maize^38^. Zhang *et al*. observed that coexistence of multiple gene actions cause oil rape improvement in Brassica^39^. Similarly, in our study, we also identified one important transcription factor WRI1 and lipid related genes showing additive mode in hybrid *Tenera*, which implies heterosis regulation every node of the gene regulation network in a hybrid.

### Tissue-specific pattern variation in heterosis

By comparing the allelic expression ratios in mesocarp and endosperm of F1 hybrid plants, we were able to test for expression variation among different tissues. Lipid biosynthesis pathway genes exhibited hybrid vigor in endosperm but not in mesocarp. However, glycolysis did not show the similar pattern. The most likely explanation for the different additively and nonadditively pattern among different tissues is that there are tissue-specific effects on their relative expression levels during crossing and evolution. This finding is similar to studies of the homoeologous genes in cotton (*Gossypium hirsutum*) polyploids, in which genes derived from the A and D subgenomes show different relative expression levels among different tissue types^40^. Some people indicated that it may be due to the allele variations that caused these different regulatory expression patterns^28^.

In summary, heterosis as a complex phenomenon, involves numerous component phenotypes, including quantitative and qualitative variations. Our data provides a key explanation to dissect the genetic basis of heterosis for oil yield in hybrid *Tenera*, comparing with its parental *Pisifera* and *Dura*. By combining lipid and transcriptome profiling, an additive expression pattern, including overdominant, dominant and nondominant effect may be the major manifestation of heterosis for oil yield in *Tenera*. Further work should address these selection networks for hybrid in a system level.

## Methods

### Oil palm plant material and field data collection

Mother palm population *Dura* (*Deli*), father palm population *Pisifera* (TS3, AVROS) and the crossing population *Tenera* oil palm (*Elaeis guineensis*) trees used in this research were planted in plantation of Wilmar International, South Sumatra (Palembang, Indonesia). The management and field yield data of the three populations was carried out following the standard protocol of Wilmar International. *Dura* fruits were harvested from trees from a *Dura* parent of Deli origin (*Dura* A), within the same self-progeny of a single tree. *Pisifera* fruits were harvested from trees from a *Tenera* X *Pisifera* parent of TS3 (African AVROS origin) within the same self-progeny of a selfed population. For each stage of development for *Dura* and *Pisifera*, at three independent bunches were tagged and harvested on three distinct individuals of the same genotype. For *Tenera* samples, two bunches of each developmental stage fruits of the tree #53 were tagged. Ten spikelets were then collected from each bunch and all undamaged fruits withdrawn from each of the ten spikelets were randomly sampled. Mesocarp and endosperm of each fruit were separated and flash frozen in liquid nitrogen.

### Bioinformatics and transcriptome data analyses

Twenty-four RNA libraries were prepared and sequenced by Illumina HiSeq2000, following the manufacturer’s instructions. Around 20 million RNAseq reads of 101 bp length were generated from mesocarp and kernel samples respectively. The quality of the generated sequence was checked by FASTQC [http://www.bioinformatics.babraham.ac.uk/projects/fastqc/]. Trinity^41^ was used for de novo assembly of the raw reads to generate unigenes. Totally 59,078 unigenes was got with around 96,062 isoforms [Supplementary Data Set S1. The *Arabidopsis thaliana* protein annotation database (TAIR 10) was used to the function annotation, with at least one hit in BLASTX search with E-value < = 1e^−3^. RSEM (RNA-Seq by Expectation Maximization)^42^ was used for abundance estimation for assembled transcripts to measure the expression level.. Information on data files containing function annotation and temporal expression is provided in Supplementary Data Set S1. ANOVA-test was used to statistical analysis for the additive and non-additive expression between different groups.

### Scanning Electron Microscopy (SEM) and light microscopy

Dry Arabidopsis seeds were collected and visualized under a scanning electron microscope (JSM-6360LV, JEOL, Tokyo, Japan). For Light microscopy, mesocarp and endosperm discs were excised from fresh oil palm fruit and fixed overnight in 2.5% glutaraldehyde in 0.1 M phosphate buffer, pH 7.2 as described before^43^. The sample discs were rinsed three times in 0.1 M phosphate buffer for 15 min each, and were then post-fixed in 1% (w/v) aqueous OsO_4_ for 1 hr. Tissues were dehydrated in an ethanol series and embedded in Spurr’s resin. Semi-thin sections with thickness of 500 nm were stained in 0.1% toluidine blue and photographed with a Zeiss Axioplan2 microscope (Carl Zeiss, Germany).

### Transgenic plasmids construction, transgenic Arabidopsis lines generation and growth conditions

The pBA-002 and pCAMBIA1300-derived vectors were used for all vector construction in this report^44^. The complete open reading frame of *EgFATB1*, *EgFATB2*, *EgDGAT1*, *EgDGAT2*, *EgWRI1*-*1*, and *EgWRI1*-*3* was amplified by PCR using the primer pairs list in Supplementary Table S4. We used *Arabidopsis thaliana* wild type (Col-0) and homozygous Arabidopsis *wri1*-*4* mutants (Col-0 background) as start plant materials. Sterilized seeds were incubated on Murashige and Skoog medium at 4 °C for 3 d and transferred to long-day conditions for growth (16 h of L/8 h of D). Transgenes were introduced into plants by *Agrobacterium tumefaciens*–mediated infiltration using the Arabidopsis floral dip method^45^. For seed mass analysis, seed mass of each plant was determined by weighing mature dry seeds collected from one whole plant.

### Lipid content analysis

To analyse the lipid content in oil palm, individual tissue and each stage of development, lipid analyses were performed in triplicate (from three different extractions) using a completely random experimental design. Each extraction is from materials of at least 3 fruits samples. The dry mesocarp and endosperm part were grinded to fine powder and the lipids were extracted with hexane three times. The weight of the total lipid was determined and the total lipid content was recorded as the ratio of total lipid to dried endosperm or mesocarp weight (lipid content, %). Total lipid content in one year for one tree was calculated as (lipid content, %) * average year endosperm or mesocarp yield per tree (Kg, based on the yield data of trees of 4th and 5th years after germination). The data were presented as means ± standard deviations.

For Arabidopsis seeds fatty acid analysis, total lipid was extracted and transmethylated from 100 dry Arabidopsis seeds as Li *et al*. described^46^. FAMEs were generated as described before^47^ and separated by GC and detected using GC Agilent 7890 coupled with 5975 C MS (Agilent, Santa Clara, CA, USA). The data were presented as means ± standard deviations.

### RNA extraction, quantitative RT-PCR and RNAseq analysis

Total RNA was extracted from all kinds of samples by grinding to fine powder in liquid nitorigen and extracted with RNeasy plant mini kits (Qiagen) or plant RNA purification reagent (Invitrogen, Carlsbad, CA, USA), following with DNAse treatment^48^. Quantitative RT-PCR for the genes in this study (Supplementary Table S6) was performed as method described before^49^. *Ubiquitin* was used as an internal control to normalize gene expression levels. Primers were listed in Supplementary Table S6.

TruSeq RNA Sample Preparation Kits v2 was used to generating mRNA-focused libraries from total RNA according to the protocol instruction.

### Accession numbers

The RNA-seq data supporting this study are available in the DNA Data Bank of Japan (DDBJ: http://www.ddbj.nig.ac.jp/) under accession number DRA001857

## Electronic supplementary material

Supplementary Figure

Supplementary Data Set
