# Author Correction: Transcriptome and functional analysis reveals hybrid vigor for oil biosynthesis in oil palm

**DOI:** 10.1038/s41598-018-23082-2

**Published:** 2018-10-25

**Authors:** Jingjing Jin, Yanwei Sun, Jing Qu, Rahmad syah, Chin-Huat Lim, Yuzer Alfiko, Nur Estya Bte Rahman, Antonius Suwanto, Genhua Yue, Limsoon Wong, Nam-Hai Chua, Jian Ye

**Affiliations:** 1 Temasek Life Sciences Laboratory, National University of Singapore, 1 Research Link, 117604 NUS, Singapore; 2 Laboratory of Plant Molecular Biology, Rockefeller University, 1230 York Avenue, New York, NY 10021 USA; 3 School of Computing, National University of Singapore, 117417 NUS, Singapore; 4 China Tobacco Gene Research Center, Zhengzhou Tobacco Research Institute of CNTC, Zhengzhou, Henan 450001 China; 5 State Key laboratory of Plant Genomics, Institute of Microbiology, Chinese Academy of Sciences, Beijing, 100101 China; 6 R&D Department, Wilmar International Plantation, Palembang, Indonesia Biotech Lab, Wilmar International, Jakarta, Indonesia

Correction to: *Scientific Reports* 10.1038/s41598-017-00438-8, published online 27 March 2017

The original version of this Article contained a typographical error in the spelling of the author Nur Estya Bte Rahman, which was incorrectly given as NurEstyaBte Rahman.

In addition Nur Estya Bte Rahman was incorrectly listed as being affiliated with ‘R&D Department, Wilmar International Plantation, Palembang, Indonesia Biotech Lab, Wilmar International, Jakarta, Indonesia’. The correct affiliation is listed below:

Temasek Life Sciences Laboratory, National University of Singapore, 1 Research Link, 117604, NUS, Singapore.

These errors have now been corrected in both the PDF and HTML versions of the Article.

