# Supplementary material for: Transcriptome and functional analysis reveals hybrid vigor for oil biosynthesis in oil palm
Source: Sci Rep. 2017 Mar 27;7:439. doi: 10.1038/s41598-017-00438-8 (PMC5428490; doi:10.1038/s41598-017-00438-8)
Supplement: Supplementary file 1 — Supplementary Figure [file 41598_2017_438_MOESM1_ESM.pdf]

Transcriptome and functional analysis reveals hybrid vigor for oil biosynthesis in oil palm

Jingjing Jin[1,2,3,6,#], Yanwei Sun[4,#], Jing Qu[1], Rahmadsyah[5], Chin-Huat Lim[5], Yuzer Alfiko[5], NurEstyaBte Rahman[5], Antonius Suwanto[5], Genhua Yue[1], Limsoon Wong[3], Nam-Hai Chua[2] and Jian Ye[1,4*]

[1]Temasek Life Sciences Laboratory, National University of Singapore, 1 Research Link, 117604 Singapore;

[2]Laboratory of Plant Molecular Biology, Rockefeller University, 1230 York Avenue, New York, NY 10021, USA;

[3]School of Computing, National University of Singapore, 117417 Singapore;

[4]State Key laboratory of Plant Genomics, Institute of Microbiology, Chinese Academy of Sciences, Beijing, 100101, China;

[5]R & D Department, Wilmar International Plantation, Palembang, Indonesia Biotech Lab, Wilmar International, Jakarta, Indonesia;

[6]China Tobacco Gene Research Center, Zhengzhou Tobacco Research Institute of CNTC, Zhengzhou, Henan, 450001, China;

[#] These authors contributed equally.

[*] Corresponding author

E-mail: jianye@im.ac.cn;

Telephone: 86-10-64807363;

Fax: 86-10-64858245

Supplementary Figure S1

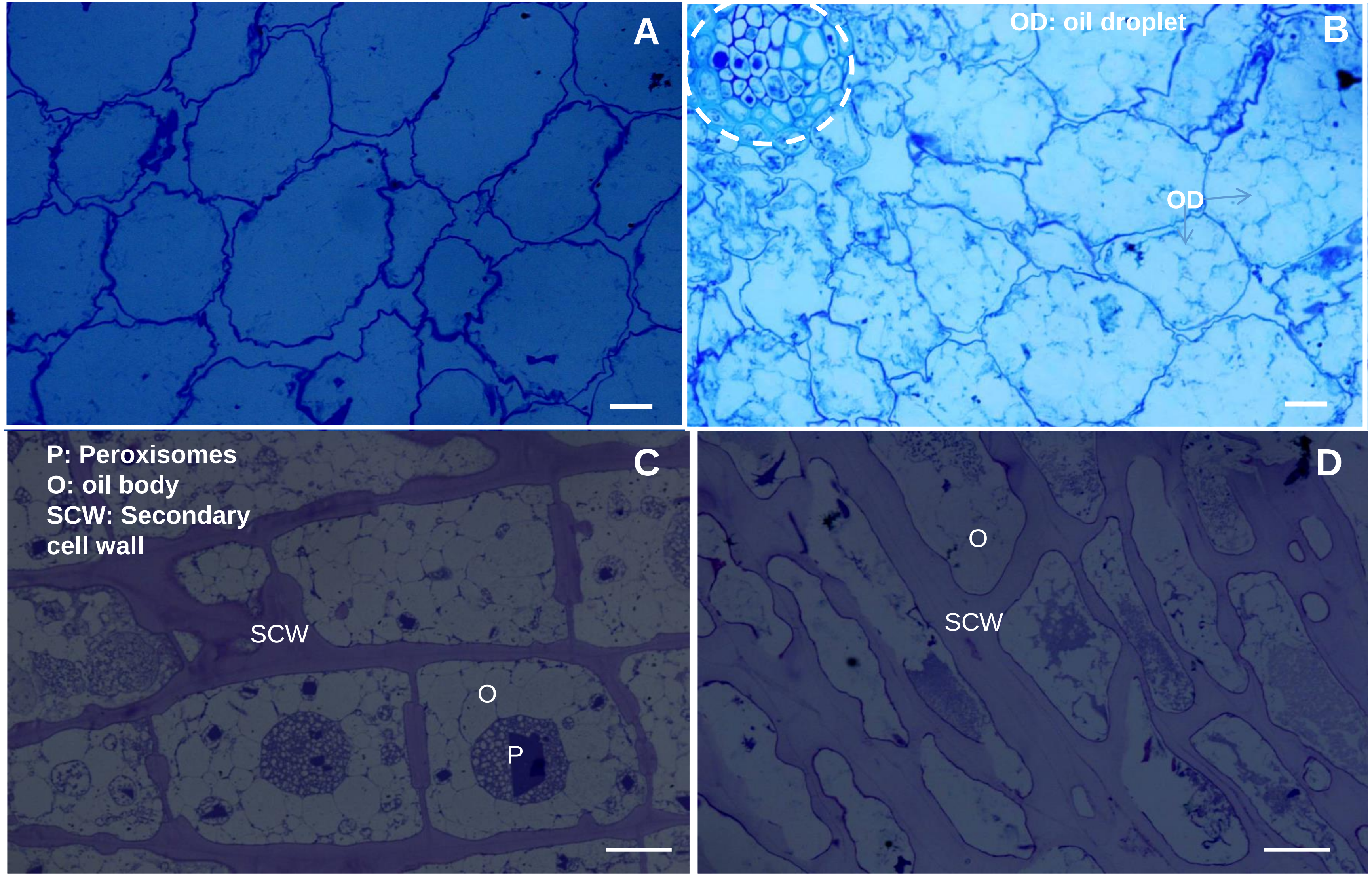

Supplementalry Figure S1: Microscopy observation of mesocarp (A and B) endosperm (C and D) and of developing oil palm fruits.
Mesocarp of *Dura* (A) and *Tenera* (B) at the same developing stage (4.5 Months After fertilization) were analysed. Broken cycle line indicates the vascular-like structure. Oil droplets (ODs) were more obvious in mesocarp of *Tenera* compared with the *Dura* mesocarp of the same developing stage. Endosperm of *Dura* in 3.5 Months After fertilization (C) and 5.5 Months After fertilization (D) were analysed. The secondary cell wall were enhanced in later stage (C) compared with early stage (D). Bar: 20µm.

Supplementary Figure S2

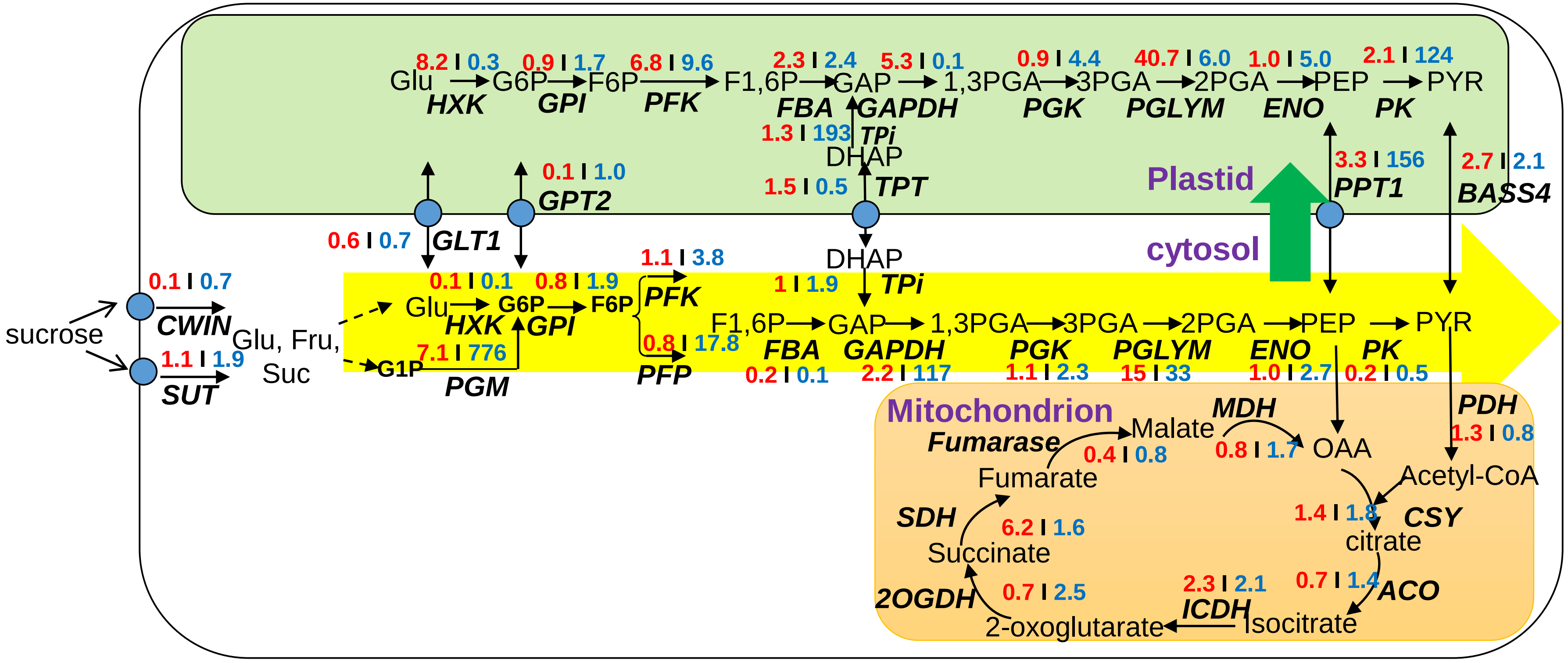

Supplementary Figure S2: Transcripts patterns for enzymes involved in glysolysis reactions for Pisifera and Tenera. Values in red indicate the fold increase in reads of mesocarp of Tenera to that of Pisifera at 3.5 MAF. Values in blue indicate the fold increase in reads of endosperm of Tenera to that of Pisifera at 2.5/4.5 MAF. Samples collected from different tissues for Dura, Pisifera and Tenera (#53) were used for transcriptome analysis.

Supplementary Figure S3

Supplementary Figure S3: Transcripts patterns for enzymes involved in plastidial and extraplasidial reactions for Pisifera and Tenera. Values in red indicate the fold increase in reads of mesocarp of Tenera to that of Pisifera at 3.5 MAF. Values in blue indicate the fold increase in reads of endosperm of Tenera to that of Pisifera at 2.5/4.5 MAF. Reads for enzymes with multiple isoforms were summed.

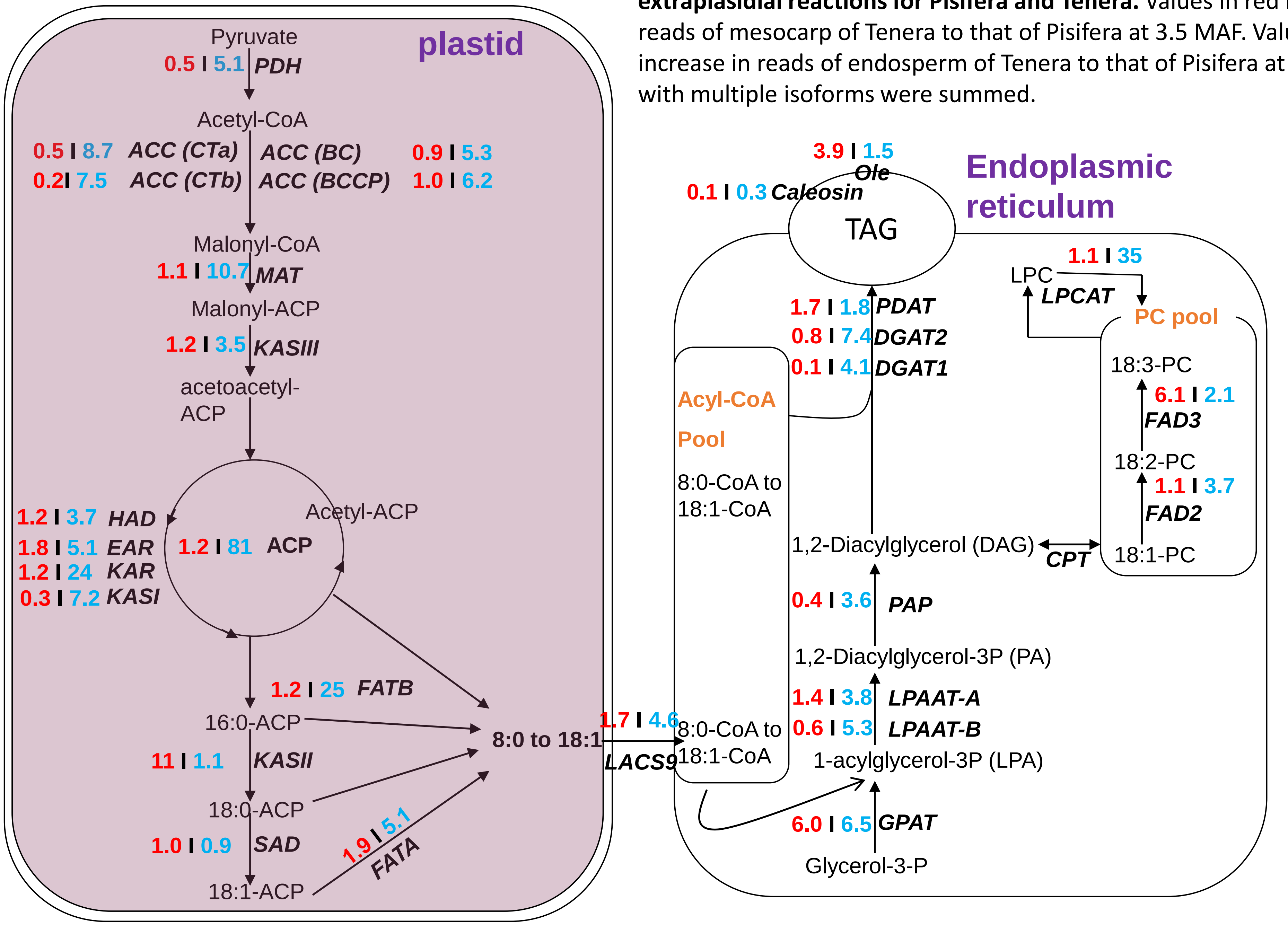

Supplementary Figure S4

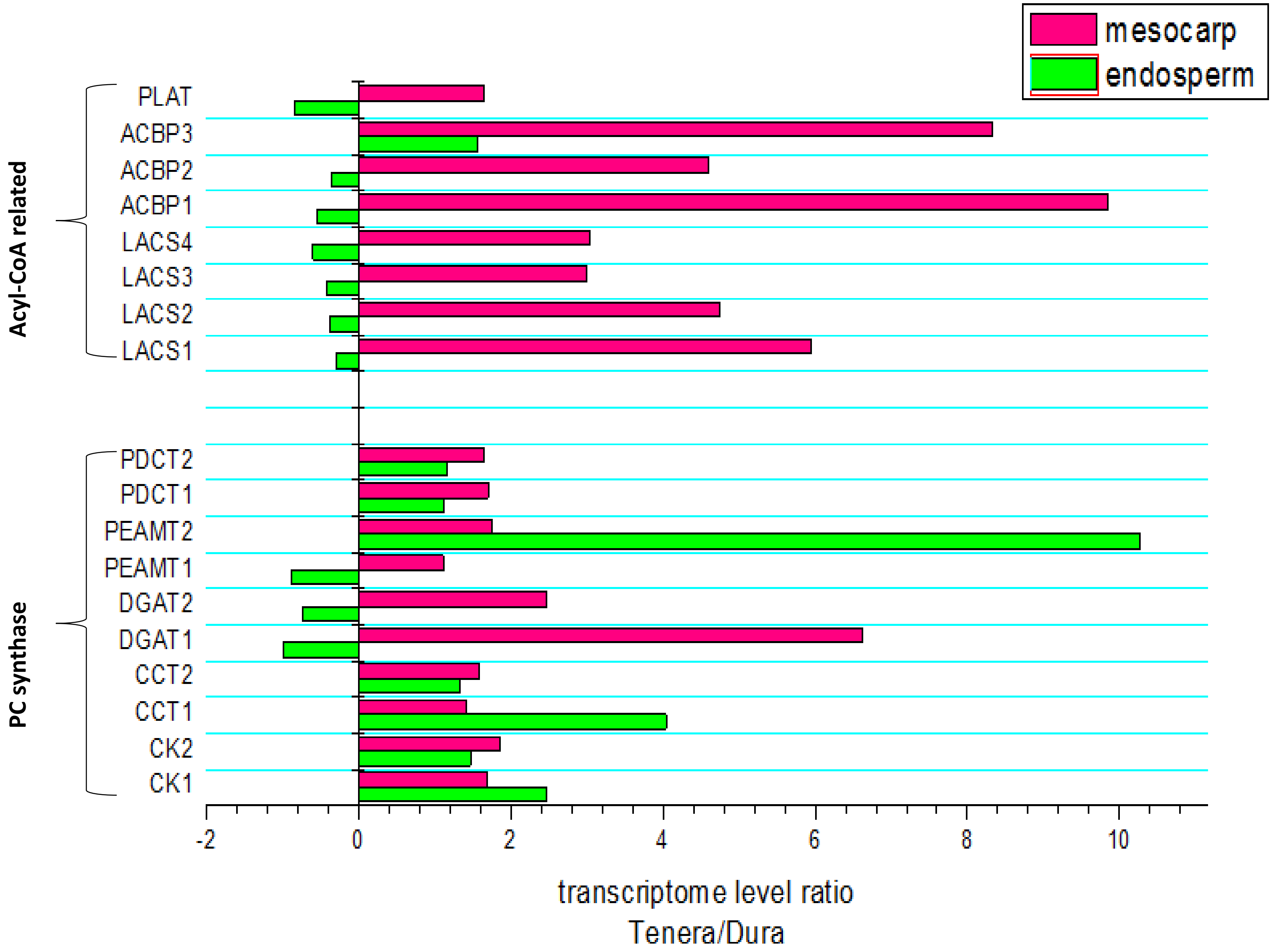

Supplementary Figure S4: Transcriptome level ratio between Tenera and Dura for PC-Related enzymes. ACBP: acyl-CoA binding protein; CCT: CTP: choline-phosphate cytidylyltransferase; CK: choline kinase; DGAT: diacylglycerol acyltransferase; LACS: long-chain acyl-CoA synthase; PDCT: phosphatidylcholine: diacylglycerol cytidylyltransferase; PEAMT: phosphoethanolamine methyl transferase; PLAT, phospholipid acyl transferase-like;

Supplementary Figure S5

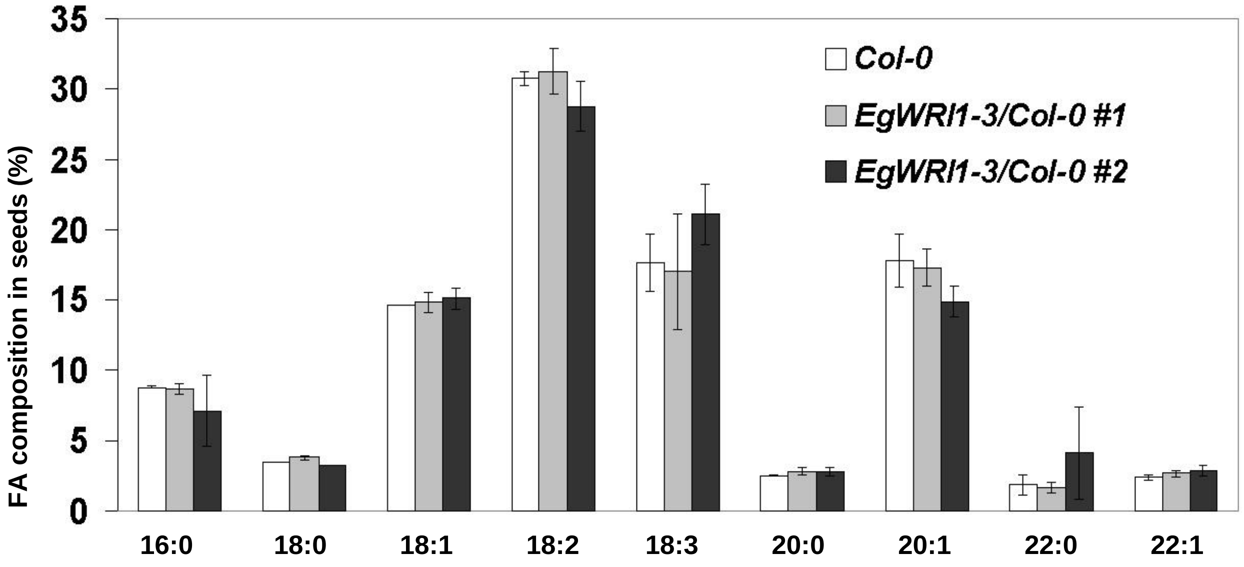

Supplementary Figure S5: Fatty Acid (FA) composition was analysed for Col-0 and two *EgWRI1-3* expression lines in WT background.

Supplementary Figure S6

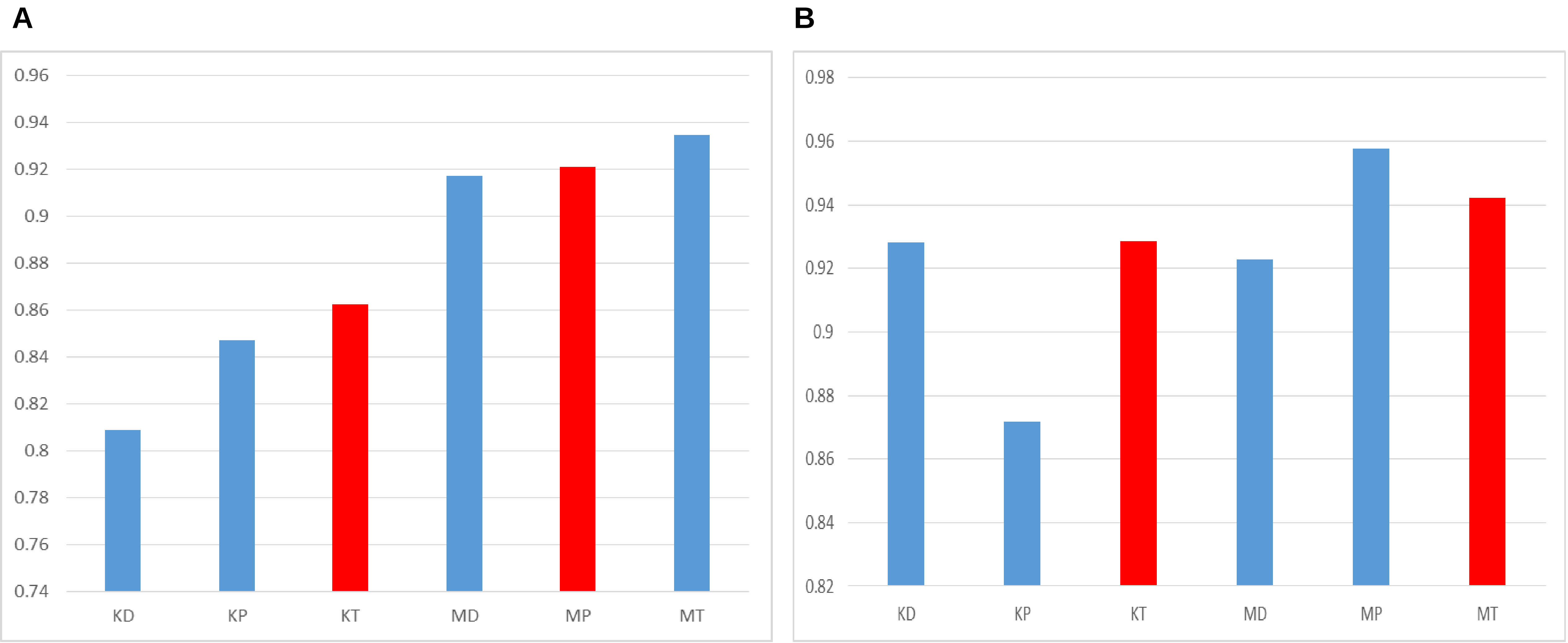

Supplementary Figure S6: Portion of allele specific genes in each group.
(A): Portion of allele specific genes based on whole gene list. **(B):** Portion of allele specific genes based on fatty acid gene list.
KD represents endosperm of *Dura*, KP represents endosperm of *Pisifera*, KT represents endosperm of *Tenera*, MD represents mesocarp for *Dura*, MP represents mesocarp of *Pisifera*, MT represents mesocarp of *Tenera*.

Supplementary Figure S7

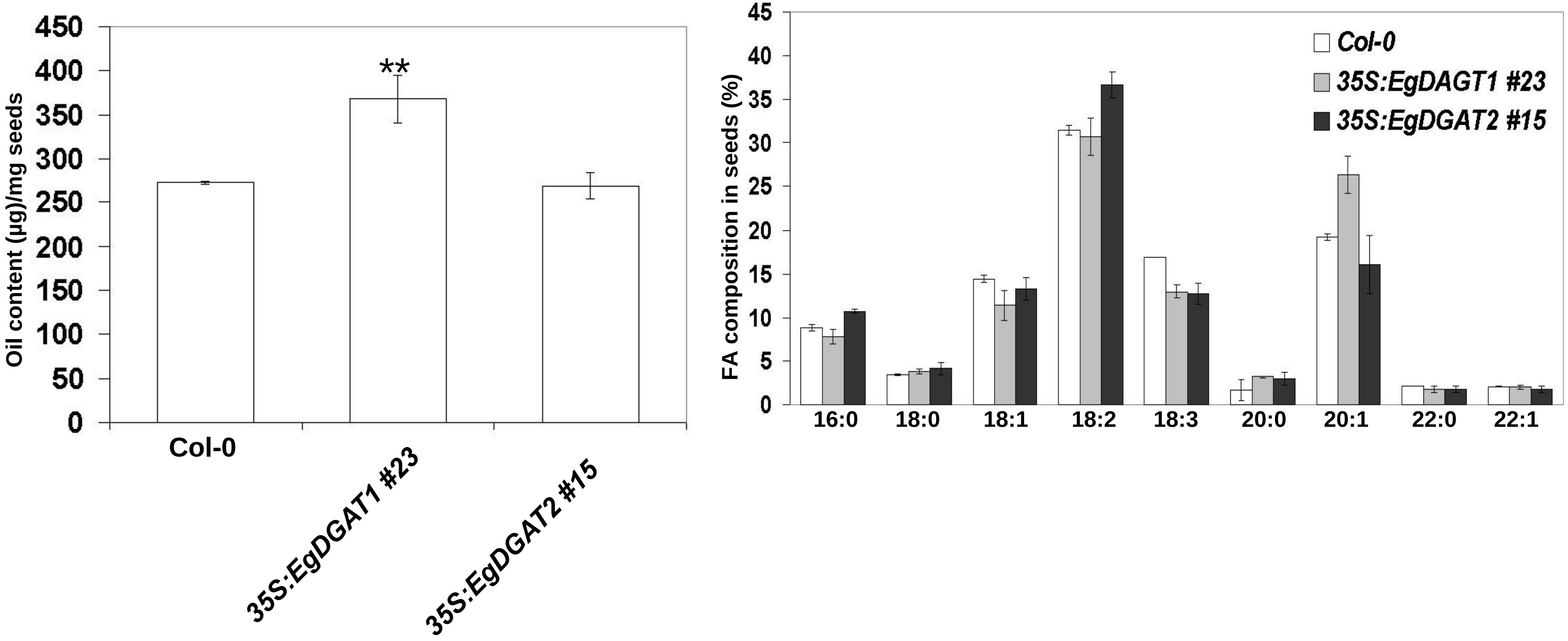

Supplementary Figure S7: Functional characterization of *EgDGAT1* and *EgDGAT2* in Arabidopsis.
The expression level of oil palm *DAGT* paralog is similar in these transgenic lines.
(A): Oil content analysis for *EgDGAT1* and *EgDGAT2* expression lines *in* Arabidopsis.
(B): Fatty Acid (FA) composition was analysed for Col-0, *EgDGAT1* and *EgDAGT2* expression lines in WT background.

Supplementary Figure S8

A

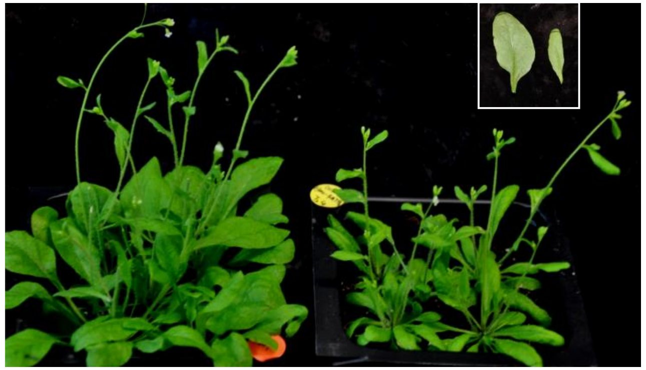

Col-0 *35S:EgFATB1 #2*

B

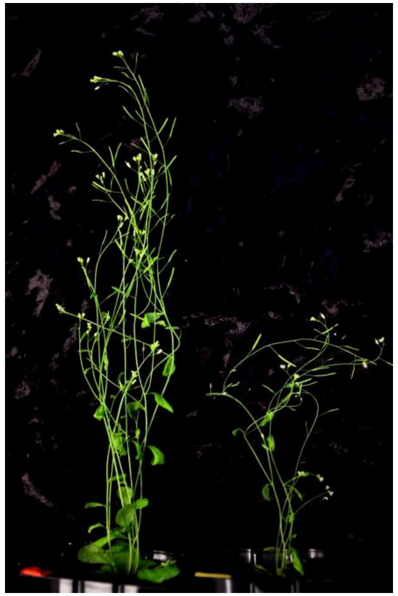

Col-0 *35S:EgFATB1 #2*

C

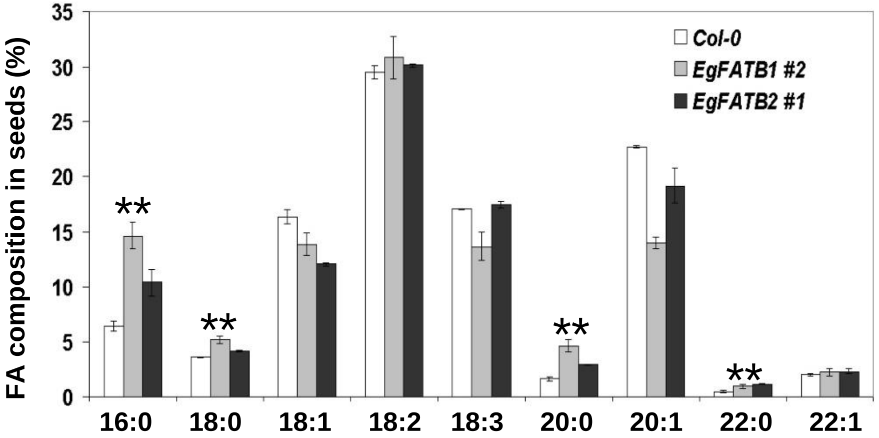

Supplementary Figure S8: Functional characterization of *EgFATB1* and *EgFATB2* in Arabidopsis.

(A and B): Phenotypes of EgFATB1 expression in Arabidopsis. Size bar:10mm.

(C): Fatty Acid (FA) composition was analysed for Col-0, *EgFATB1* and *EgFATB2* expression lines in WT background. The expression level of oil palm *FATB* paralog is similar in these transgenic lines.

Supplementary Table S1

#Term	Database	ID	P-Value
colanic acid building blocks biosynthesis	BioCyc	COLANSYN-PWY	0.002560546
choline biosynthesis I	BioCyc	PWY-3385	0.003959964
DNA replication	KEGG PATHWAY	ath03030	0.007049678
**Mannose metabolism**	**PANTHER**	**P02752**	**0.007566702**
**Fatty acid metabolism**	**KEGG PATHWAY**	**ath01212**	**0.009768289**
**phospholipid biosynthesis II**	**BioCyc**	**PHOSLIPSYN2-PWY**	**0.011253982**
**xylan biosynthesis**	**BioCyc**	**PWY-5800**	**0.012080739**
Amino sugar and nucleotide sugar metabolism	KEGG PATHWAY	ath00520	0.015341161
**phosphatidylethanolamine biosynthesis II**	**BioCyc**	**PWY4FS-6**	**0.017648576**
superpathway of choline biosynthesis	BioCyc	PWY-4762	0.01874096
**Fatty acid biosynthesis**	**KEGG PATHWAY**	**ath00061**	**0.018867899**
Fructose and mannose metabolism	KEGG PATHWAY	ath00051	0.023247125
Protein export	KEGG PATHWAY	ath03060	0.025432248
Phagosome	KEGG PATHWAY	ath04145	0.026473654
**superpathway of fatty acid biosynthesis II (plant)**	**BioCyc**	**PWY-5156**	**0.027667005**
**GDP-mannose biosynthesis**	**BioCyc**	**PWY-5659**	**0.02965234**
**fatty acid biosynthesis initiation I**	**BioCyc**	**PWY-4381**	**0.02965234**
**sphingolipid biosynthesis (plants)**	**BioCyc**	**PWY-5129**	**0.031534486**
**cellulose biosynthesis**	**BioCyc**	**PWY-1001**	**0.036814426**
**GDP-glucose biosynthesis**	**BioCyc**	**PWY-5661**	**0.036814426**
**UDP-glucose biosynthesis (from glucose 6-phosphate)**	**BioCyc**	**PWYQT-4437**	**0.04498809**
Protein processing in endoplasmic reticulum	KEGG PATHWAY	ath04141	0.045155965
**Glycerophospholipid metabolism**	**KEGG PATHWAY**	**ath00564**	**0.045665184**
**fatty acid beta-oxidation II (peroxisome)**	**BioCyc**	**PWY-5136**	**0.046299876**
Purine metabolism	KEGG PATHWAY	ath00230	0.047628854

Supplementary Table S1: Enriched pathway by gene set in class 5 and 6 of Table 1 for endosperm.

Supplementary Table S2

#Term	Database	ID	P-Value
**superpathway of carotenoid biosynthesis**	**BioCyc**	**CAROTENOID-PWY**	**0.0001227**
**cytokinins-O-glucoside biosynthesis**	BioCyc	PWY-2902	0.0071702
**Carotenoid biosynthesis**	**KEGG PATHWAY**	**ath00906**	**0.0103129**
methionine biosynthesis II	BioCyc	PWY-702	0.0115112
**Photosynthesis**	KEGG PATHWAY	ath00195	0.019201
**lutein biosynthesis**	**BioCyc**	**PWY-5947**	**0.0263469**
salvianin biosynthesis	BioCyc	PWY-5268	0.0263469
**trans-lycopene biosynthesis II (plants)**	**BioCyc**	**PWY-6475**	**0.0263469**
shisonin biosynthesis	BioCyc	PWY-5284	0.0263469
Pentose phosphate pathway	KEGG PATHWAY	ath00030	0.0309417
pelargonidin conjugates biosynthesis	BioCyc	PWY-5139	0.0342649
Metabolic pathways	KEGG PATHWAY	ath01100	0.0392
Selenocompound metabolism	KEGG PATHWAY	ath00450	0.0484728

Supplementary Table S2: Enriched pathway by gene set in class 5 and 6 of Table 1 for mesocarp.

Supplementary Table S3

		Dura	*Pisifera*	*Tenera*
1.5 MAF	C14:0	0±0	0±0	0±0
	C16:0	41.8±2.0	46.2±0.6 *	42.8±5.7
	C18:0	9.4±0.5	10.4±0.4 *	7.3±1.4*
	C18:1	13.1±0.5	13.4±2.5	12.1±1.3
	C18:2	27.1±1.5	26.5±2.2	33.2±3.0 *
	C18:3	7.0±0.3	3.6±0.5*	4.6±1.2*
	others	1.5±0.2	0±0*	0±0*
2.5 MAF	C14:0	1.3±0.9	0.5±0*	2.1±0.2
	C16:0	42.0±1.0	47.2±0.1*	39.1±2.0
	C18:0	9.7±0.5	7.0±0.5*	8.2±1.3
	C18:1	21.4±1.1	32.7±0.8*	19.0±0.8*
	C18:2	19.9±1.2	23.1±0.1*	24.2±0.5*
	C18:3	5.3±0.5	3.6±0.2*	6.4±0.3*
	others	0.3±0.1	0±0*	1.1±0.6*
3.5 MAF	C14:0	0.9±0.1	0.6±0.1*	1.50±0.2*
	C16:0	42.6±2.3	44.8±0.6	38.3±1.0*
	C18:0	6.5±0.3	4.7±0.2*	5.4±0.1*
	C18:1	35.4±3.2	32.7±0.8	34.5±0.6
	C18:2	12.5±1.1	17.1±0.4*	16.7±0.9*
	C18:3	0.4±0	0.3±0.1	2.1±0.1*
	others	0.3±0	0±0*	1.5±0.6*
4.5 MAF	C14:0	1.1±0.1	1.2±0	1.7±0*
	C16:0	43.7±3.2	45.3±0.6	54.3±1.8*
	C18:0	4.0±0.2	5.1±0.1*	3.6±0.4
	C18:1	43.3±2.1	30.8±2.1*	33.4±2.7*
	C18:2	7.7±1.2	17.4±0.6*	7.0±0
	C18:3	0.3±0	0.3±0	0.2±0*
	others	0±0	0±0	0±0
5.5 MAF	C14:0	1.3±0.1	0.6±0.1*	2.0±0.0*
	C16:0	42.9±1.2	41.3±1.1	45.6±0.5*
	C18:0	5.8±0.2	4.8±0.7*	4.3±0.1*
	C18:1	40.2±0.6	38.6±0.8*	38.0±0.1*
	C18:2	9.6±1.6	14.7±1.8*	9.5±0.3
	C18:3	0.2±0.1	0.2±0	0.3±0
	others	0±0	0±0	0±0

Supplementary Table S3: Fatty acid composition (mol%) of the developing endosperm of oil palm (months after fertilization, MAF). Data was presented as average of 3 fruit repeats ± SD. The significance of the effect of the varieties was tested by t-Test (P values, *, P<0.05) when compared between *Dura* with either *Pisifera* or *Tenera*.

Supplementary Table S4

	FA	*Dura*	*Pisifera*	*Tenera*
1.5 MAF	C8:0	0±0	nd	0±0
	C10:0	0±00	nd	0±0
	C12:0	0.9±0.2	nd	0.1±0*
	C14:0	0.6±0.1	nd	1.6±0.6*
	C16:0	34.6±3.6	nd	38.2±5.2
	C18:0	6.3±0.6	nd	4.3±0.9*
	C18:1	45.8±5.3	nd	41.9±6.2
	C18:2	11.4±1.6	nd	13.6±0.6
	C18:3	0.3±0.1	nd	0.3±0.1
2.5 MAF	C8:0	0.2±0.1	0±0*	0.3±0.1
	C10:0	2.3±0.2	3.9±0.2*	3.0±0.6
	C12:0	30.2±2.0	55.9±3.6*	32.1±2.8
	C14:0	12.3±1.9	20.0±1.4*	10.6±1.3
	C16:0	14.3±0.8	7.8±0.3*	13.7±0.5
	C18:0	2.5±0.6	2.6±0.6	3.2±0.3
	C18:1	30.9±1.2	7.7±1.2*	30.3±3.5
	C18:2	7.0±0.2	1.8±0.4*	6.6±0.6
	C18:3	0.3±0.1	0.2±0.1	0.2±0.1
3.5 MAF	C8:0	0.3±0.1	0±0*	0.6±0.5*
	C10:0	5.1±0.8	3.0±0.6*	4.5±0.7
	C12:0	58.2±4.8	50.2±5.1	50.9±6.3
	C14:0	15.7±1.1	16.9±2.1	18.8±1.5
	C16:0	6.7±1.2	8.5±0.8	8.0±1.6
	C18:0	1.6±0.3	2.2±0.3	1.3±0.3
	C18:1	10.6±2.6	15.8±1.3*	12.8±3.1
	C18:2	2.0±0.5	3.5±0.4*	2.8±0.7
	C18:3	0.1±0	0.2±0*	0.2±0.1
4.5 MAF	C8:0	0.2±0	0±0	1.1±0.6*
	C10:0	4.7±0	2.4±0.5*	5.0±0.4
	C12:0	57.3±0.3	50.7±3.1*	56.1±2.1
	C14:0	16.5±0.3	19.3±1.6*	17.7±0.3*
	C16:0	6.9±0.1	9.8±0.4*	6.5±0.5
	C18:0	1.6±0	1.3±0.2*	1.3±0.1
	C18:1	11.3±0.1	13.5±0.6*	10.6±0.9
	C18:2	1.7±0	2.7±0.1*	1.5±0*
	C18:3	0.1±0	0.2±0.1	0.2±0*
5.5 MAF	C8:0	0.2±0	0.9±0.2*	0±0*
	C10:0	4.4±0.1	4.4±0.7	4.1±0.2
	C12:0	51.9±0.2	57.2±1.4*	50.0±2.0
	C14:0	18.1±0.1	17.5±0.8*	18.6±0.9
	C16:0	8.4±0.1	6.2±0.8*	8.2±0.9
	C18:0	1.8±0	1.1±0.1*	1.7±0.1
	C18:1	13.0±0.1	11.1±0.6*	13.7±0.4
	C18:2	2.4±0	1.3±0*	2.5±0.2
	C18:3	0.1±0	0.3±0.1*	0.1±0

Supplementary Table S4: Fatty acid composition (mol%) of the developing mesocarp (months after fertilization, MAF). Data was presented as average of at least 3 fruit repeats ± SD. The significance of the effect of the varieties was tested by t-Test (P values, *, P<0.05) when compared between *Dura* with either *Pisifera* or *Tenera*.

Supplementary Table S5

Tenera VS *Dura*	Total No. (UP: Down), Ratio of UP/Down in Endosperm	Total No. (UP: Down), Ratio of UP/Down in Mesocarp
**Whole transcriptome**	**59078 (3364:2789), 1.2**	**59078 (8300:616), 13.5**
**Total in FA pathway**	**338(36: 20), 1.8**	**338 (51: 1), 51**
Plastidial Fatty Acid Synthesis	57 (10: 1), **10**	57 (7: 1), **7**
TAG Synthesis	22 (1: 1), 1	22 (0: 0)
Extra Plastidial Phospholipid Synthesis	52 (11: 1), **11**	52 (8: 0)
Other Fatty Acid and TAG Synthesis Related Genes	20 (0: 2), 0	20 (3: 0)
Plastid Polar Lipids	33 (0: 2), 0	33 (12:0)
Mitochondrial Lipid Synthesis	23 (1: 2), 0.5	23 (4:0)
Sphingolipid Synthesis	42 (6:1), **6**	42 (4:0)
oleosin	4 (0: 1), 0	4 (0: 0)
Lipase	55 (5:5), 1	55 (10:0)
β-Oxidation	42 (2:4), 0.5	42 (5: 0)
**Glycolysis**	**131 (17:13), 1.3**	**131 (20:2), 10**
**Glycolysis (plastid)**	**55(5:5), 1**	**55 (7:2), 3.5**

Supplementary Table S5: Summary of up- or down-regulated genes when compared the expression level of *Tenera* against *Dura*. Assumed at least 2 folds change as up- or down-regulated genes.

Supplementary Table S6

Gene	Sequence (5’-3’)	Purpose
EgWRI1-1	AATAAGTCGACATGAAGAACTCTCCTCCCTCTACT	Plant expression
	TTAATTCTAGACTAGGCACCTTTGCTTGCACTGAAACCA	Plant expression
EgWRI1-3	AATAAGTCGACATGAAGAAATCTCCTCCCTCTCCTCC	Plant expression
	TTAATTCTAGACTAAGAGCATATACTAATCTGAAGGGGGT	Plant expression
EgDGAT1	AACAAGTCGACATGGCGGTCTCGAAGAATCCAGAAA	Plant expression
	GATCTGGATCCCTCTGTTCCCACTTTTCTGTTCAT	Plant expression
EgDGAT2	AGCGCGTCGACATGGGGGCAGAATTCTGTCTCTCTCTCT	Plant expression
	AGTCTGGATCCTAAAACTCTCAAACGAAAAT	Plant expression
EgFATB1	AGACAGTCGACATGGTTGCTTCGATTGTCGCTTGGGCCTTTTT	Plant expression
	TTATTCTAGATCAAGCACTTCCAGCTGAAGTGGGA	Plant expression
EgFATB2	AGATGTCGACATGGTTGCTTCAATTGCCGCCT	Plant expression
	TTATTCTAGATCATGCACTACCACCTGGAGTTGG	Plant expression
EgFATB3	AATACAGTCGACATGGTCGCCTCCGTTGCTGCCTC	Plant expression
	TTATTTCTAGAT$_{T}$ATCATTTAGTCTCAGTTGGGAGCA	Plant expression
EgWRI1-1-F	GTCTACAAGCATCGCCTACAG	qRT-PCR
EgWRI1-1-R	CAGGTCGTAATTTGGCATGGT	qRT-PCR
EgWRI1-2-F	ACCTTCGACAAGGACCATGA	qRT-PCR
EgWRI1-2-R	TGTCCTGTCCATGGCAGCCTG	qRT-PCR
EgWRI1-3-F	GGACACCAGGAGTTTAGCCCA	qRT-PCR
EgWRI1-3-R	ATTCTTCCATGCTAATGATAC	qRT-PCR
EgEF1F	CCTGTGCTTGATTGCCAC	qRT-PCR
EgEF1R	GTCTCAACCACCATAGGC	qRT-PCR

Supplementary Table S6: List of primers used in this study.
